# Serpin family proteins as potential biomarkers and therapeutic drugs in stroke: A systematic review and meta‐analysis on clinical/preclinical studies

**DOI:** 10.1111/cns.14205

**Published:** 2023-04-05

**Authors:** Bing Yan, Lu Luo, Li Liu, Zhenyu Wang, Ruiying Chen, Yi Wu, Xiao Xiao

**Affiliations:** ^1^ Key Laboratory of Computational Neuroscience and Brain‐Inspired Intelligence, Ministry of Education, Behavioral and Cognitive Neuroscience Center, Institute of Science and Technology for Brain‐Inspired Intelligence, MOE Frontiers Center for Brain Science Fudan University Shanghai 200433 China; ^2^ Department of Rehabilitation Medicine, Huashan Hospital Fudan University Shanghai 200040 China; ^3^ School of Life Sciences Fudan University Shanghai 200032 China

**Keywords:** antithrombin, C1‐INH, FUT175, meta‐analysis, serpin, stroke, systematic review

## Abstract

**Background:**

Serpin is a superfamily of serine proteinase inhibitors. They have anticoagulative activities and immunoregulatory effects. The family has been widely studied in stroke patients and animal stroke models. However, results from clinical and preclinical studies are controversial. The systematic review and meta‐analysis aimed to determine whether serpin activities are affected by stroke and whether members of the serpin family could be used in stroke treatment.

**Methods:**

Literature was systematically searched in six databases until September 5, 2022. In the included studies, 47 clinical studies (8276 subjects) reported concentrations of serpin proteins in stroke patients and healthy controls. In total, 41 preclinical studies (742 animals) reported neurological outcomes in animal models with serpin treatment and vehicle.

**Results:**

Meta‐analysis of clinical studies showed that both ischemic (IS) and hemorrhagic stroke patients had higher thrombin‐antithrombin complex (TAT) levels and lower antithrombin (AT) levels which were persistent in the acute and subacute phase of IS. Meta‐analysis of preclinical studies reported the efficacy of serpins in treating stroke. C1‐INH and FUT175 reduced brain infarct size and improved sensorimotor and motor behavior in a dose‐ and time‐dependent manner in the MCAO models.

**Conclusions:**

Our study confirmed the important roles serpin family proteins played in the onset, progression, and treatment of stroke. Among serpins, AT and TAT may be used as blood biomarkers in the early diagnosis of stroke. C1‐INH and FUT175 could be potential medications for IS.

## Graphical abstract

The human serpin antithrombin and its complex with thrombin, in combination with other circulating factors, are novel blood biomarkers in the early diagnosis of stroke. Preclinical studies have consolidated the therapeutic effects of C1‐INH and FUT175 in treating ischemic stroke, which act in a dose‐ and time‐dependent manner.

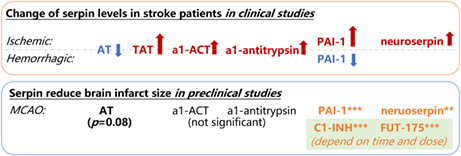

## 1 INTRODUCTION

Stroke is a global burden. It ranks fifth among all causes of death.^1^ It leads to serious long‐term disability and complications in survivors, such as depression,^2^ cognitive impairment, and dementia.^1^ Ischemic stroke (IS) is more frequent worldwide while hemorrhagic stroke (HS) causes more death proportionally.^1^, ^3^ Intravenous tissue‐type plasminogen activator (tPA) is a proven intervention for acute IS. However, the therapeutic benefit largely depends on the onset‐to‐treatment (OTT) time.^4^, ^5^ A timely tPA treatment needs improvement as only a minority of patients receive it within the 4.5‐h OTT window.^6^ Endovascular treatment is the other guideline‐recommended intervention for the early management of acute IS.^7^, ^8^, ^9^ However, a lack of complete reperfusion is observed at the end of thrombectomy in most patients.^10^ Both intravenous thrombolysis and endovascular therapy have a narrow treatment window and a risk of hemorrhagic transformation.^11^

Serpin is a superfamily of serine proteinase inhibitors with typical primary structure.^12^, ^13^ In total, 34 serpins have been identified in the human body and divided into nine clades.^13^ Many human serpins have anticoagulative and antifibrinolytic activities. For example, antithrombin (AT, SERPINC1) primarily targets the coagulant and anticoagulant substances such as thrombin and Factor Xa and regulates hemostasis and coagulation.^14^ Plasminogen activator inhibitor‐1 (PAI‐1, SERPINE1) regulates the plasminogen system by rapidly inhibiting tPA and the urokinase‐type plasminogen activator.^15^ Serpins also have immunoregulatory and anti‐inflammatory signaling effects. For example, AT has a potent cardioprotective effect via activating the adenosine monophosphate‐activated protein kinase signaling pathway.^14^, ^16^ The primary role of α1‐antitrypsin (SERPINA1) is to inhibit the neutrophil elastase and protect tissues from its attack.^17^ C1 esterase inhibitor (C1‐INH, SERPING1) regulates the classical complement pathway and the MBL pathway by inhibiting C1s/C1r and MASP‐1/MASP‐2, respectively.^18^ C1‐INH has been used in treating hereditary angioedema.^19^ The synthetic serpin Nafamostat mesilate (FUT175) also has potent activity against the classical complement pathway and the alternative pathway.^20^, ^21^ It has been clinically used as an anticoagulant.^22^, ^23^ Preclinical studies have validated its antitumor^24^ and cardioprotective effects.^25^ The functions of serpin proteins have been widely studied in cardiovascular disease, cancer, and metabolic disturbances.^15^, ^26^

Serpin family proteins share substrates in the anticoagulative/inflammatory system but may behave differently in stroke. The anticoagulative and anti‐inflammatory activities of serpin proteins make them potential markers/regulators in the diagnosis/treatment of stroke. However, clinical studies reported altered serpin levels in stroke patients with controversial results. Meanwhile, preclinical studies have tested serpin as a treatment for stroke using animal models. This systematic review and meta‐analysis aim at summarizing evidence on serpin and stroke to investigate (1) if serpin proteins act as blood biomarkers of stroke and (2) if serpin proteins have therapeutic effects on stroke.

## 2 METHOD

### 2.1 Literature search

**FIGURE 1:**
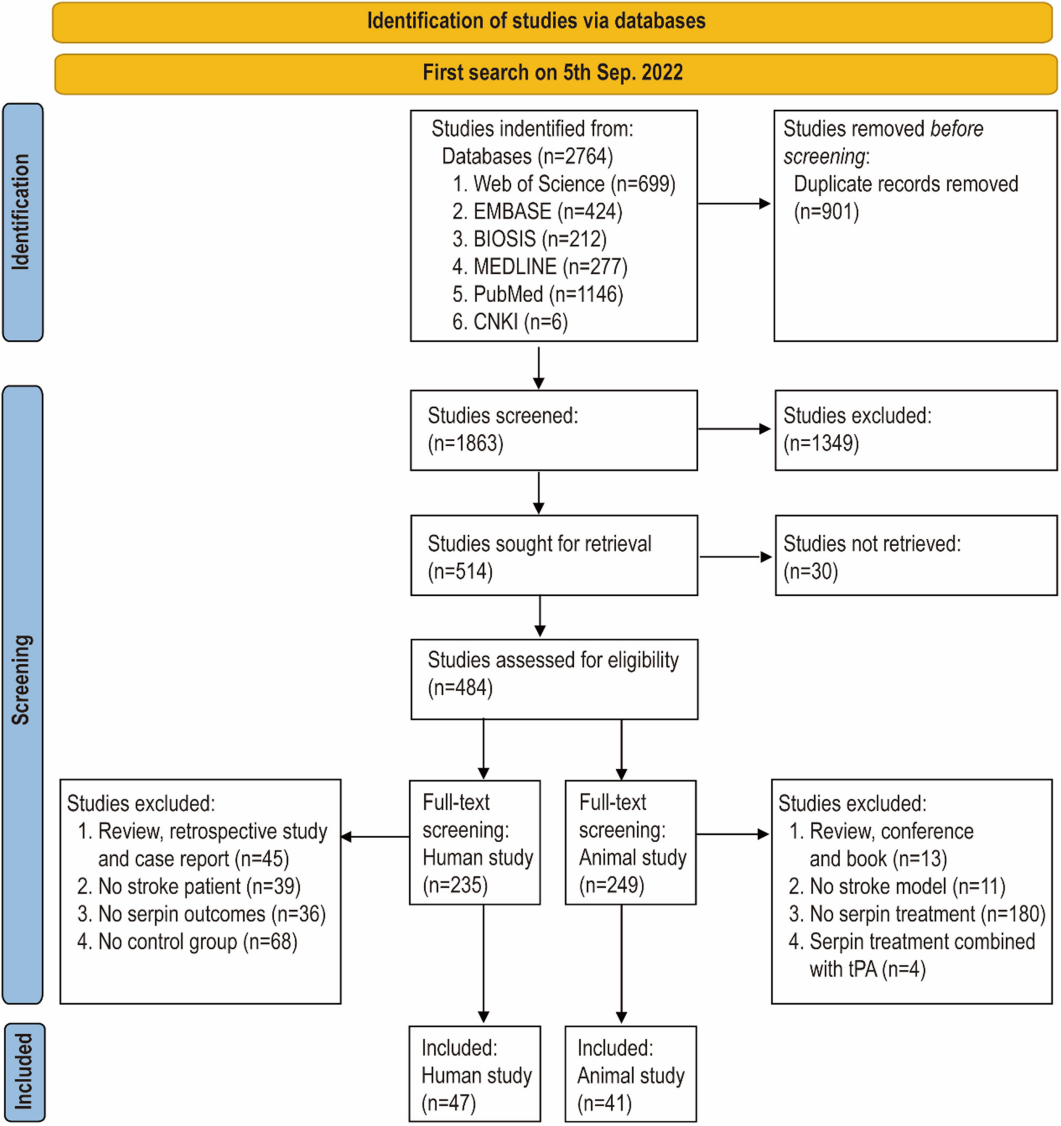
PRISMA flow chart.

This systematic review and meta‐analysis were registered on PROSPERO for clinical studies (CRD42021268081) and preclinical studies (CRD42021268063). It has followed the guides to systematically identify all relevant animal studies^27^, ^28^ and reported by the Preferred Reporting Items for Systematic Reviews and Meta‐Analysis (PRISMA) statement.^29^ The literature search was performed in six bibliographic databases, including Web of Science, EMBASE, BIOSIS, MEDLINE, PubMed, and China National Knowledge Infrastructure (CNKI) until September 5, 2022. Keywords relative to stroke and serpin were used (Supplementary 1 in Appendix S1). In total, 2764 studies were found in the literature search. After removing duplicates, 1863 studies went through title/abstract screening and full‐text screening. The literature search and screening were performed by two review authors independently. Any disagreements were resolved through discussion with a third reviewer. In total, 47 clinical studies reporting serpin concentrations in stroke patients and healthy people were included. In total, 41 preclinical studies reporting the therapeutic effects of serpins in animal models of stroke were included (Figure 1).

### 2.2 Classification of stroke and animal models

In clinical studies, stroke was categorized into IS, HS, and transient ischemic attack (TIA). IS had five major subtypes according to the Trial of ORG 10172 in Acute Stroke Treatment (TOAST) classification system, including the large artery disease, the cardiac embolism, the small‐vessel occlusion (lacunar), the stroke of undetermined etiology (cryptogenic) and the stroke of other determined etiology.^30^, ^31^ Patients reported as cerebral infarction, cerebral thrombosis, cerebral embolism, atherothrombotic, and atherosclerotic IS were included in IS as well. Subtypes of HS included subarachnoid hemorrhage (SAH) and intracerebral hemorrhage (ICH). The time course of stroke was divided into the acute phase (<7 days), the subacute phase (7 days–6 months), and the chronic phase (>6 months).

In preclinical studies, animal models of IS included the middle cerebral artery occlusion (MCAO), hypoxic–ischemic (HI), and photothrombosis. The MCAO model, which was most widely used, could be further divided into transient (tMACO) and permanent type (pMCAO) depending on the duration of occlusion. Animal models of HS included SAH and ICH.

### 2.3 Study inclusion/exclusion criteria

In full‐text screening, potentially eligible studies were split into clinical studies and preclinical studies. They were screened separately according to the inclusion and exclusion criteria. To prevent bias, inclusion criteria of clinical studies were pre‐specified as follows: (1) population: patients with stroke (including all ages/sexes) and no comorbidity with other diseases; (2) intervention: no treatment; (3) control: healthy people without intervention; (4) outcome: concentrations of serpin proteins in the blood/serum/plasma tested by ELISA or relative RNA levels of serpins tested by real‐time quantitative PCR. Pre‐specified exclusion criteria were: (1) patients with no stroke, stroke patients with co‐morbidities; (2) case studies, cross‐over studies, studies without a separate control group; (3) stroke patients who had received treatment; (4) no control group as healthy people; (5) no outcomes of serpin levels.

For preclinical studies, pre‐specified inclusion criteria were: (1) population: animal models of stroke (including all species/sexes), in controlled studies with one or more separate control groups; (2) intervention: administration of serpin, including all serpin family proteins and all administration routes; (3) control: vehicle (e.g., saline or artificial cerebrospinal fluid); (4) outcome: neurological outcomes such as brain infarct size and behavioral scores/tests. Pre‐specified exclusion criteria were: (1) no animal models, animals with co‐morbidities, in silico models; (2) case studies, cross‐over studies, studies without a separate control group; (3) studies with no serpin treatment, studies with combined treatment of serpin and tPA; (4) no vehicle group as control; (5) no neurological outcomes.

### 2.4 Data extraction

In clinical studies, data were extracted as (1) study characteristics, including author name, year published, and study design; (2) participant characteristics, including the number of participants, mean age, gender, and type of disease; (3) outcomes, including the serpin concentrations in protein level and mRNA level.

In preclinical studies, the following data were extracted: (1) study characteristics; (2) subject characteristics, including the number of animals, species, age, gender, weight, and type of model; (3) intervention, including the type of serpin, dosage, injection method, and timepoint; (4) two continuous outcomes, including the mean and variance of (i) infarct size (in mm^3^ or %) measured by 2,3,5‐Triphenyltetrazolium chloride (TTC) staining and other immunohistochemical methods and (ii) behavioral scores (e.g., Bederson score, focal neurological score) and motor and sensorimotor performance measured by behavioral tests (e.g., grip test and limb‐placing test).

Data were extracted by two review authors independently. When the numerical values of outcomes were not available directly from texts and tables in the published paper, an email would be sent to contact the corresponding author for inquiry. After two attempts with no replies received, outcomes were measured from graphs in the published paper using WebPlotDigitizer (Version 4.3) by two authors independently.^32^ It was accepted if the deviation of extracted data by two authors was no more than 20%. Then mean values of the extracted data by two authors were used in the meta‐analysis. Otherwise, two authors performed the measurement again.

### 2.5 Quality assessment

Risk of bias assessment was performed according to the Cochrane RoB tool for clinical studies^33^ and the SYRCLE's RoB tool for preclinical studies^34^ by two review authors independently. Any discrepancies were discussed and resolved by consensus. The assessment was performed using RevMan 5.4.1.

### 2.6 Data analysis

Stata/IC 17.0 (StataCorp) was used to perform the meta‐analysis. An adjusted standardized mean difference (Hedges's *g*) was used as the measure of effect size.^35^ Random‐effects model was used to calculate the overall effect.^36^ The heterogeneity of the experiments included in each group was analyzed based on Cochrane's *Q* and reported as *I* ^2^.^37^

In the overall analysis of clinical studies, experiments were divided into three groups according to stroke types, including IS, HS, and TIA. If serpin levels at different timepoints were reported, the outcome at the first timepoint was used in the analysis. In the time‐course analysis, the disease course of IS was divided into acute phase (<7 days), subacute phase (7 days–6 months), and chronic phase (>6 months). Outcomes reported at the first timepoint in each phase were used in the analysis.

In preclinical studies, the overall effect on reducing brain infarct size was calculated for each serpin in three animal models of IS, including MCAO, HI, and photothrombosis. When more than one treatment group (e.g., different administration routes of serpin) and one control group were reported in a single study, each was considered as an independent experiment and the number of animals in the control group was split to avoid repeated comparison.^38^ When multiple dosages and timepoints of administration were investigated in one study, the highest dose/earliest timepoint was used in the overall analysis. Subgroup analyses of dosage were performed in studies of FUT175 and C1‐INH separately. Between‐group difference was calculated based on the homogeneity test of Cochran's *Q*. A recommended significance level of 0.1 (#*p* < 0.1) was used instead of the more conservational level of 0.05.^39^ Meta‐regression analysis of the therapeutic effect and serpin dosage at several timepoints was performed to reveal the dose‐ and time‐dependent effect.

### 2.7 Publication bias and sensitivity analysis

Funnel plot and Egger regression‐based test were used to analyze publication bias and small‐study effects.^40^ To assess the effect of low‐quality studies and the robustness of meta‐analysis, sensitivity analysis was performed by the leave‐one‐out method. A single study was omitted at a time and the overall effect was analyzed on the remaining studies.

## 3 RESULTS

### 3.1 Clinical evidence: AT and TAT are potential blood biomarkers of IS/HS

#### 3.1.1 Study characteristics and quality

In total, 47 clinical studies reporting outcomes of serpin levels in stroke patients and healthy people were identified (Supplementary 2 in Appendix S1). In total, 4905 stroke patients (4497 patients with IS, 320 with HS and 88 with TIA) and 3371 healthy controls were included in the meta‐analysis. Clinical studies reported outcomes of PAI‐1 (*n* = 20), AT (*n* = 12), thrombin‐antithrombin complex (TAT, *n* = 15), α1‐antitrypsin (*n* = 5), α1‐antichymotrypsin (α1‐ACT, SERPINA3, *n* = 1), vaspin (SERPINA12, *n* = 3), neuroserpin (SERPINI1, *n* = 1), the FVIIa‐antithrombin complex (FVIIa‐AT, *n* = 1), and the pseudogene SERPINB9P1 (*n* = 1). 95% of studies had a low risk of bias in incomplete outcome data (Supplementary 3 in Appendix S1). More than 90% of studies had high selection bias and performance bias because they were not double‐blinded randomized controlled clinical trials.

#### 3.1.2 Overall and time‐course change

**FIGURE 2:**
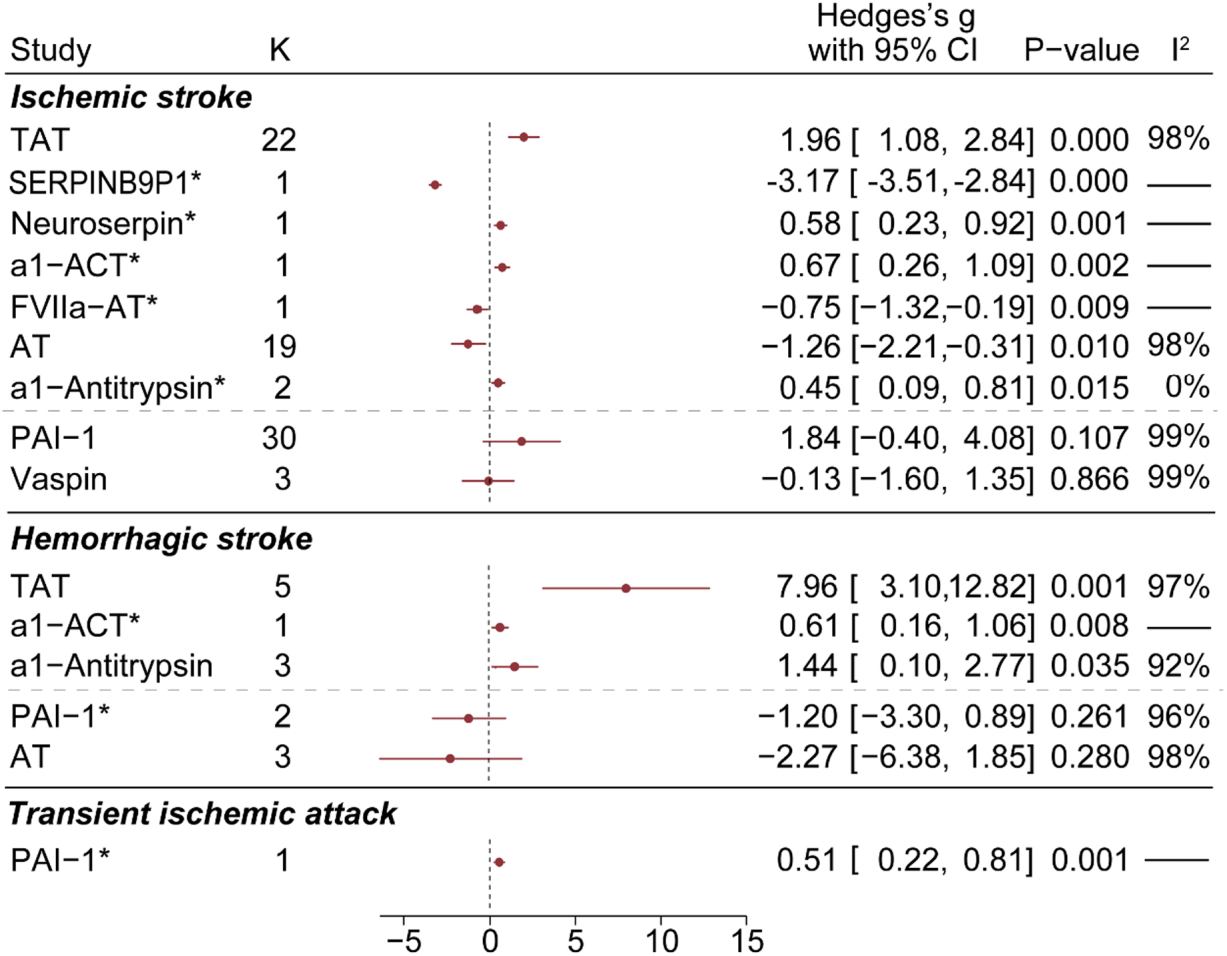
Serpin levels are affected in stroke patients. Serpin concentrations were compared between stroke patients and healthy people. Results were sorted by *p*‐value. *K* indicated the number of experiments pooled in each group. Red dot and lines represented SMD (Hedges's *g*) with 95% CI. *p*‐Value was calculated from *z*‐test. *I* ^2^ indicated heterogeneity of studies in each group. Asterisk (*) following serpin name indicated that the number of experiments was no more than 3. Horizontal dashed lines separated serpins with significant effect size (*p* < 0.05) and those without.

The concentration of each serpin protein in stroke patients was compared to healthy people (Figure 2). The blood level of AT was lower in IS (SMD = −1.26 [95% CI, −2.21 to −0.31]; *p* < 0.05) and HS patients (SMD = −2.27 [95% CI, −6.38 to 1.85]; *p* = 0.28) while the TAT level was higher in both IS (SMD = 1.96 [95% CI, 1.08 to 2.84]; *p* < 0.001) and HS patients (SMD = 7.96 [95% CI, 3.10 to 12.82]; *p* < 0.01). In contrast, the PAI‐1 level was higher in ischemic conditions (IS: SMD = 1.84 [95% CI, −0.40 to 4.08]; *p* = 0.107; TIA: SMD = 0.51 [95% CI, 0.22 to 0.81]; *p* < 0.01) and lower in HS patients (SMD = −1.20 [95% CI, −3.30 to 0.89]; *p* = 0.261). IS and HS patients had slightly higher levels of a1‐ACT and a1‐antitrypsin, the change of which were much milder as compared to TAT. IS patients had more neuroserpin (SMD = 0.58 [95% CI, 0.23 to 0.92]; *p* < 0.01) and less FVIIa‐AT (SMD = −0.75 [95% CI, −1.32 to −0.19]; *p* < 0.01) and vaspin (SMD = −0.13 [95% CI, −1.60 to 1.35]; *p* = 0.866). The long non‐coding RNA level of pseudogene SERPINB9P1 was reported as downregulated in IS patients in one study (SMD = −3.17 [95% CI, −3.51 to −2.84]; *p* < 0.001).

**FIGURE 3:**
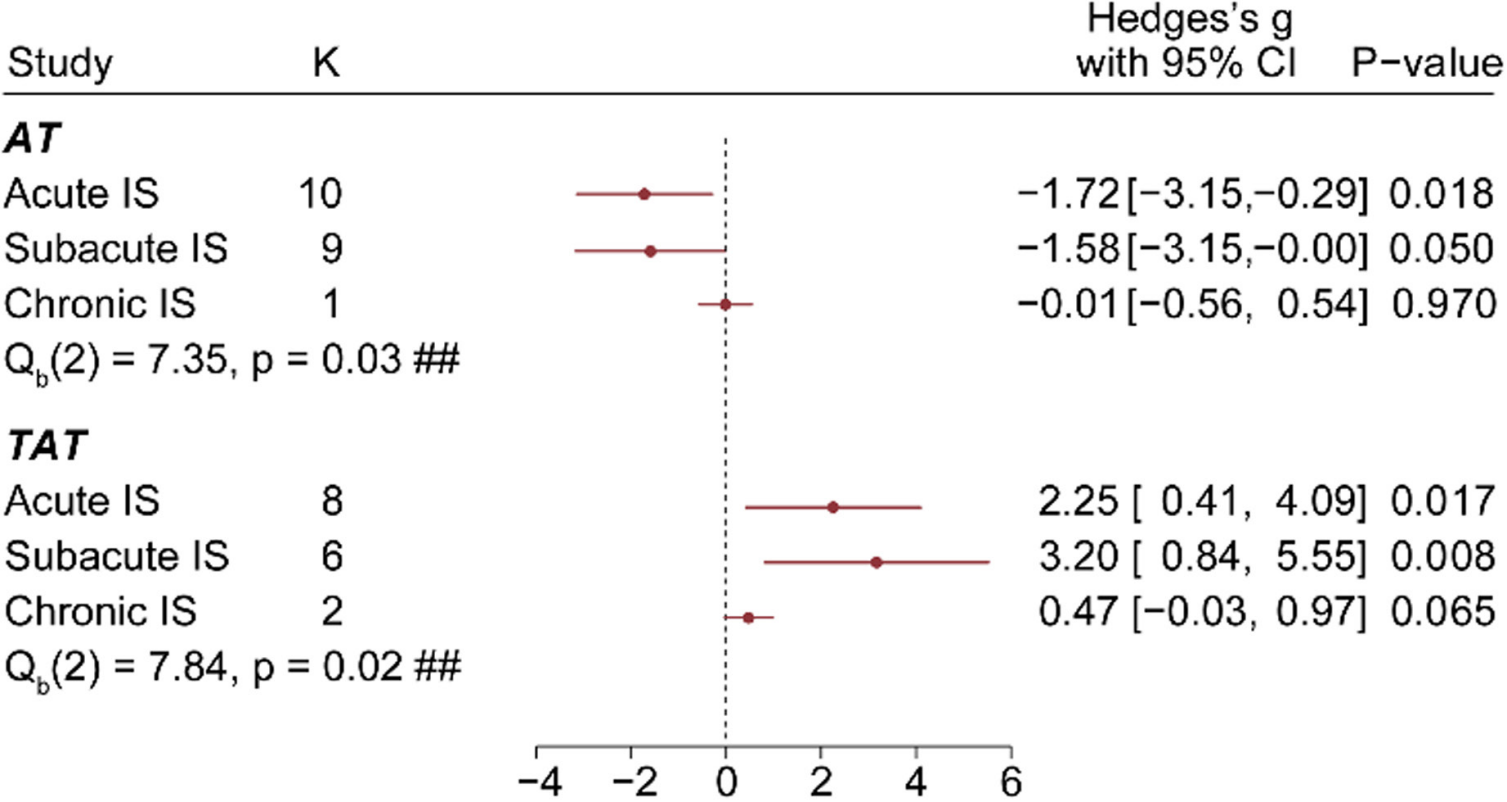
Change in AT and TAT levels in three phases of ischemic stroke. Patients of ischemic stroke were divided into three groups according to disease progression, including the acute phase (<7 days), the subacute phase (7 days–6 months), and the chronic phase (>6 month). Between‐group difference ## *p* < 0.05.

In different phases of IS, the lower AT level and higher TAT level observed above were consistent from the acute phase (<7 days) to the subacute phase (7 days–1 month; Figure 3). In the chronic phase of IS, both AT and TAT dropped back to the normal level with fewer experiments included (*n* = 1 or 2).

#### 3.1.3 Publication bias and sensitivity analysis

Publication bias was assessed in the studies reporting PAI‐1, AT, and TAT (Supplementary 4 in Appendix S1). Small‐study effects were observed in the HS group of AT (*z* = −3.48, *p* < 0.001) and TAT (*z* = 4.46, *p* < 0.001), and the IS group of PAI‐1 (*z* = 11.19, *p* < 0.001), AT (*z* = −3.67, *p* < 0.001), and TAT (*z* = 3.83, *p* < 0.001). Leave‐one‐out analysis was performed to test the robustness of our findings. When a single study was removed, the overall effect of the remaining studies fell into the range of 95% CI of the original result (Supplementary 5 in Appendix S1).

### 3.2 Preclinical evidence: C1‐INH and FUT175 have therapeutic effects on IS

#### 3.2.1 Study characteristics and quality

In total, 41 studies of serpins in treating animal models of stroke were identified in the literature search. Nine human serpins including C1‐INH (*n* = 10), PAI‐1 (*n* = 6), PEDF (SERPINF1, *n* = 5), neuroserpin (*n* = 4), AT (*n* = 2), α1‐antitrypsin (*n* = 1), α1‐ACT (*n* = 1), LEX032 (SERPINA3, *n* = 1), PAI‐2 (SERPINB2, *n* = 1), two serpin artifacts including FUT175 (*n* = 7) and Aprotinin (*n* = 2), and one viral serpin CrmA (SERPINN, *n* = 1) were reported (Supplementary 6 in Appendix S1). The brain infarct size was used as the neurological outcome to estimate the treatment effect of serpin. In total, 10 studies did not report the brain infarct size and therefore were excluded in the following meta‐analysis of overall estimation. 95% of studies had a low risk of bias in Baseline characteristics (Supplementary 7 in Appendix S1). Many studies had low‐performance bias, detection bias, and attribution bias.

#### 3.2.2 Overall effect of serpin on infarct size in IS models

**FIGURE 4:**
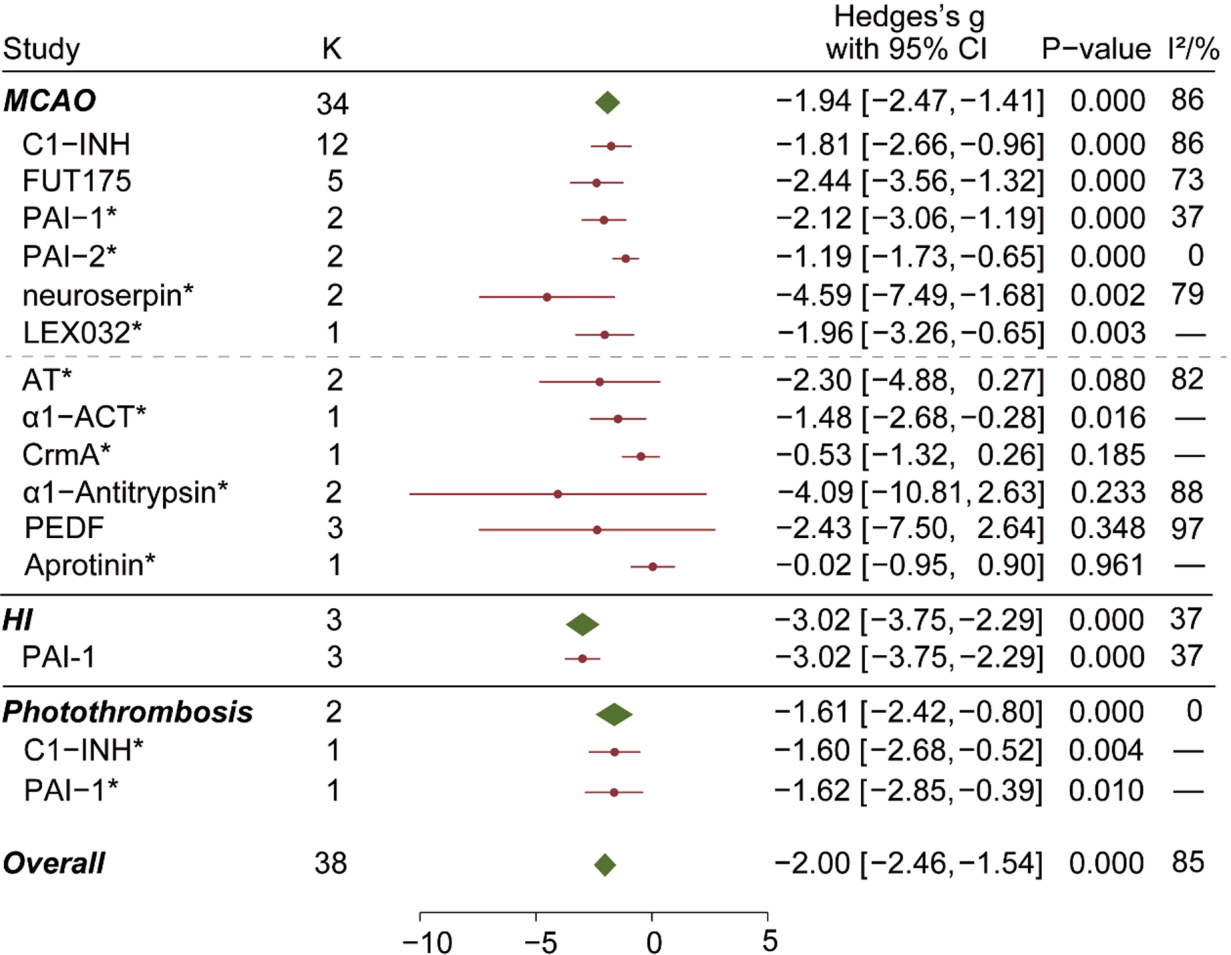
Overall effect of serpins on brain infarct size in IS animal models. Forest plot showing the therapeutic effect of serpins in reducing infarct size. C1‐INH, C1 esterase inhibitor; PEDF, pigment epithelium‐derived factor. *K* indicated the number of experiments pooled in each group. Red dot and lines represented SMD (Hedges's *g*) with 95% CI. *p*‐Value was calculated from *z*‐test. *I* ^2^ indicated heterogeneity of studies in each group. Asterisk (*) following serpin name indicated that the number of experiments was no more than 3. Horizontal dashed lines separated serpins with significant effect size (*p* < 0.05) and those without.

In total, 742 animals from 31 experiments were included in the overall analysis of serpin efficacy (Figure 4). In the MCAO models, brain infarct size was significantly reduced by C1‐INH (SMD = −1.81 [95% CI, −2.66 to −0.96]; *p* < 0.001) and FUT175 (SMD = −2.44 [95% CI, −3.56 to −1.32]; *p* < 0.001). Besides, C1‐INH reduced infarct size in the photothrombosis model as well (SMD = −1.60 [95% CI, −2.68 to −0.52]; *p* < 0.01). The high heterogeneity in studies of C1‐INH (*I* ^2^ = 86%) and FUT175 (*I* ^2^ = 73%) could arise from the variety of drug dosages and injection timepoints, which were further analyzed below.

Similarly, the brain infarct size in animals with MCAO was reduced by PAI‐1 (*p* < 0.001), PAI‐2 (*p* < 0.001), neuroserpin (*p* < 0.01), and LEX032 (*p* < 0.01), as reported in a small number of studies. Among them, PAI‐1 was reported effective in other ischemic models including HI (SMD = −3.02 [95% CI, −3.75 to −2.29]; *p* < 0.001) and photothrombosis (SMD = −1.62, [95% CI, −2.85 to −0.39]; *p* < 0.05). AT reduced the infarct size in MCAO animals by the effect size of −2.30 (95% CI, −4.88 to 0.27; *p* = 0.08). As compared to AT, a1‐ACT, crmA, and aprotinin had relatively smaller effects. α1‐antitrypsin and PEDF had relatively larger effects but higher variance within the group.

#### 3.2.3 Dose‐dependent effect of FUT175 in MCAO models

**FIGURE 5:**
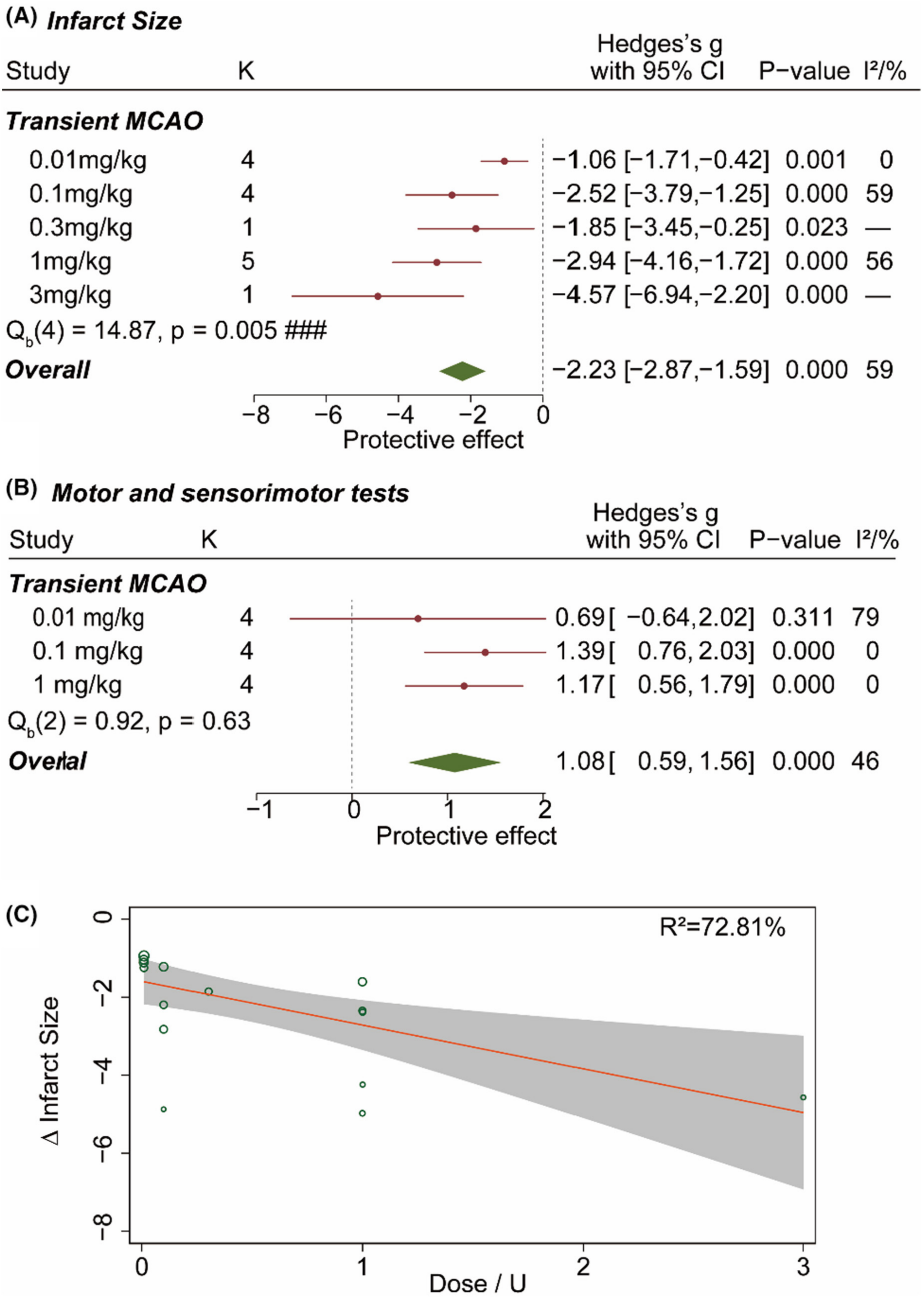
Does‐dependent effect of FUT175 in the tMCAO model. (A) Dose‐dependent effect of FUT175 on brain infarct size. ### *p* < 0.01. (B) Dose‐dependent effect of FUT175 on motor and sensorimotor ability. Hedges's *g* above 0 indicated improved behavior and protective effect. (C) Meta‐regression analysis on the effect of FUT175 on brain infarct size. FUT175 dosage was used as the mediator. There was a correlation between the reduced infarct size and FUT175 dosage.

In total, 170 animals from 15 experiments were included in the subgroup analysis of FUT175 dosage. The FUT175 drug used in the preclinical study was manufactured by Nanjing R&D Pharma and Torii Pharma mainly. Brain infarct size was effectively reduced by a range of dosage from 0.01 mg/kg (SMD = −1.06 [95% CI, −1.71 to −0.42]; *p* < 0.01), 0.1 mg/kg (SMD = −2.52 [95% CI, −3.79 to −1.25]; *p* < 0.001) to 1 mg/kg (SMD = −2.94 [95% CI, −4.16 to −1.72]; *p* < 0.001; Figure 5A). The motor and sensorimotor ability of animals with stroke was improved by FUT175 at 0.1 mg/kg (SMD = 1.39 [95% CI, 0.76 to 2.03]; *p* < 0.001) and 1 mg/kg (SMD = 1.17 [95% CI, 0.56 to 1.79]; *p* < 0.001; Figure 5B). Meta‐regression analysis further supported that FUT175 reduced infarct size in tMCAO animals in a dose‐dependent manner (*R* ^2^ = 73%; Figure 5C).

#### 3.2.4 Dose‐ and time‐dependent effect of C1‐INH in MCAO models

**FIGURE 6:**
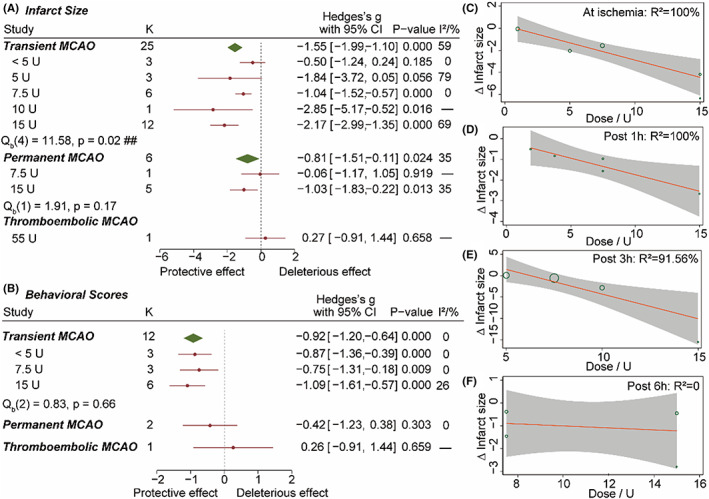
Dose‐ and time‐dependent effect of C1‐INH in IS animal models. (A) Dose‐dependent effect of C1‐INH on brain infarct size. ## *p* < 0.05. (B) Dose‐dependent effect of C1‐INH on behavioral scores. Hedges's *g* below 0 indicated improved behavior and protective effect. (C–F) Meta‐regression analysis on the effect of C1‐INH on brain infarct size in transient MCAO model. The C1‐INH dosage was used as the mediator. There was a correlation between the therapeutic efficacy and dosage when C1‐INH was injected at the beginning of ischemia (C), post 1 h (D) and post 3 h (E) after reperfusion. No correlation was found when C1‐INH was injected post 6 h after reperfusion (F).

In total, 431 animals from 32 experiments were included in the subgroup analysis of C1‐INH. The drug C1‐INH tested in the preclinical study was manufactured by CSL Behring, Baxter‐Immuno, and Pharming. In the tMCAO model, administration of 1–5U C1‐INH improved the composite behavioral scores (SMD = −0.87 [95% CI, −1.36 to −0.39]; *p* < 0.001; Figure 6B) but did not significantly reduce infarct size (*p* = 0.185; Figure 6A). 7.5U C1‐INH effectively reduced infarct size (SMD = −1.04 [95% CI, −1.52 to −0.57]; *p* < 0.001) and improved behavioral scores (SMD = −0.75 [95% CI, −1.31 to −0.18]; *p* < 0.01) in the tMCAO model, but did not reduce infarct size in the pMCAO model (*p* = 0.919, number of experiments = 1). A high dose of 15U C1‐INH was effective in treating both tMCAO and pMCAO.

The heterogeneity in the subgroups of 5U (*I* ^2^ = 79%) and 15U (*I* ^2^ = 69%) could arise from the variety of administration time. Therefore, we further investigated the dose‐dependent efficacy in each group with different C1‐INH injection timepoints. In 5 experiments from 2 studies, C1‐INH was administrated post 1 h of tMCAO. The effect size of infarct size reduction was negatively correlated to dosage (*R* ^2^ = 100%; Figure 6D). A similar correlation was observed when C1‐INH was administrated post 3 h of tMCAO (*R* ^2^ = 92%, in 4 experiments from 2 studies; Figure 6E) or at the beginning of ischemia (*R* ^2^ = 100%, in 5 experiments from 3 studies; Figure 6C). However, there was no correlation at post 6 h of tMCAO (*R* ^2^ = 0%; Figure 6F). Therefore, it was inferred that the therapeutic effect of C1‐INH on the tMCAO model depended on both drug dosage and treatment timepoint relative to stroke onset. Within 3 h following tMACO onset, a higher dose of C1‐INH gave better efficacy.

#### 3.2.5 Publication bias and sensitivity analysis

Publication bias was assessed using funnel plots (Supplementary 8 in Appendix S1). There was a small‐study effect in the studies of C1‐INH in MCAO models (*z* = −3.35, *p* < 0.001). No small‐study effect was observed in studies of FUT175 (*z* = −1.51, *p* = 0.132). When a single study was removed from the sensitivity analysis, the overall effect of the remaining studies fell into the range of 95% CI of the original result (Supplementary 9 in Appendix S1).

## 4 DISCUSSION

The systematic review and meta‐analysis examined clinical and preclinical evidence of serpin family proteins in the pathogenesis and treatment of stroke. Clinical studies revealed that both IS and HS patients had decreased concentrations of AT and increased concentrations of TAT, which could act as diagnostic biomarkers. Consistently, preclinical results showed that AT effectively treated IS by reducing brain infarct size in animal models of MCAO (*p* = 0.08). AT is the primary thrombin inhibitor in the human body that prevents blood coagulation. It regulates the activity of thrombin by forming the TAT complex in endothelial cell injury and the following thrombosis. TAT is the indicator of thrombin activation and is related to cardiovascular disease progression and poor outcomes. A previous meta‐analysis reported that the increased circulating TAT level was associated with subsequent stroke in patients with atrial fibrillation.^41^ A more recent meta‐analysis showed that plasma TAT levels were higher in IS patients with different TAT levels in each subtype due to the underlying mechanism of embolus formation.^42^ Higher TAT level was associated with elevated mortality rates and poor outcomes in IS patients treated with tPA, probably due to revascularisation resistance.^43^ Besides its anticoagulant effect, AT may treat ischemic conditions without reaching the clot‐formation site by suppressing the role that thrombin directly plays in neuronal death.^44^ AT and TAT are not specific markers of stroke. For example, patients with localized cancer had elevated TAT level.^45^ Therefore, AT/TAT could be used with other circulating factors as combined blood markers in boosting the diagnostic efficiency of stroke.

A large number of studies in our meta‐analysis reported PAI‐1, which played a crucial role in the fibrinolytic system and has been recognized as the central hub in the pathogenesis and progression of thrombotic vascular diseases.^46^ Our meta‐analysis showed that PAI‐1 was increased in IS (*p* = 0.12) while decreased in HS (*p* = 0.26). Previous studies have reported that PAI‐1 level was significantly higher in IS patients with lone AF and patients with atherothrombotic large artery disease.^41^, ^47^ In our meta‐analysis, different subtypes of IS were pooled together and analyzed as the ischemic condition in comparison to the hemorrhagic condition because each IS subtype had a limited number of experiments. Therefore, the heterogeneity of IS subtypes may give rise to the insignificant results we observed in PAI‐1 change. Our preclinical study analysis showed that PAI‐1 effectively reduced brain infarct size in ischemic conditions of MCAO, HI, and photothrombosis. However, there were contradictory findings in previous studies using the stroke model of PAI‐1‐deficient mice.^48^, ^49^, ^50^ This difference may arise from the heterogeneity of animal models. PAI‐1 is the main endogenous inhibitor of tPA in the human body. As a proven intervention for IS, tPA potentiates both excitotoxic and ischemic neuronal death.^51^ Neuron‐derived tPA activates microglia at the injury site while microglia‐derived tPA mediates neurodegeneration.^52^, ^53^ PAI‐1 may inhibit the excitotoxic and neurodegenerative effect of tPA and maintain homeostasis. Likewise, our meta‐analysis showed that neuroserpin was slightly increased in IS patients (SMD = 0.58) while it reduced infarct size in MCAO animal models. Clinical reports showed that higher neuroserpin level before intervention in IS patients was associated with better functional outcomes.^54^ The decline in neuroserpin level within the first 24 h from IS onset was associated with inhibition of excitotoxicity, inflammation, and blood–brain barrier (BBB) disruption.^55^ Neuroserpin may inhibit NMDA activation and calcium accumulation in neurons induced by tPA.^56^, ^57^ The mechanism underlying its neuroprotective role is still debated, including both tPA‐dependent and tPA‐independent pathways.^58^ It helps maintain the structure of BBB, protect neurons/astrocytes, and suppress neuroinflammatory responses such as microglial activation after stroke.^59^ A recent study reported that intranasal delivery of serpina3n, the mouse homolog of human α1‐ACT, reduced infarct size and rescued neurological deficits after stroke. Similarly, it attenuated BBB disruption by suppressing immune cell infiltration into the brain.^60^ Besides, our meta‐analysis indicated that α1‐antitrypsin level was higher in both IS and HS patients though, it reduced the infarct size in MCAO models (*p* = 0.23). In human body, α1‐antitrypsin reduces the inflammatory activity of protease enzyme elastase. The genetic polymorphism of α1‐antitrypsin may play a more important role in stroke. A common coding variant [M1 (A213V)] in α1‐antitrypsin is a risk factor for large artery stroke.^61^ The M1 mutation may alter the function of α1‐antitrypsin without affecting its circulating level or in vitro enzymatic activity.^62^

In preclinical studies, our meta‐analysis summarized the therapeutic effects of C1‐INH, a human serpin, and FUT175, a synthetic serpin artifact. Both effectively reduced brain infarct size and improved motor/sensorimotor functions in animal models of MCAO. Functionally close to each other, C1‐INH and FUT175 are both complement inhibitors.^63^, ^64^ After cerebral ischemia, complement components are deposited on neurons. The deleterious inflammatory responses lead to neuronal death, especially after reperfusion.^65^ Inhibition of the complement activation after stroke onset may have a neuroprotective effect.^66^ C1‐INH suppresses complement activation by binding MBL in the lectin pathway. It also plays a vital role in the interaction among complement, coagulation, and fibrinolytic systems.^67^ Meanwhile, it could function in a complement‐independent way as it reduced reperfusion injury in the C1q^−/−^ mice.^68^ Clinically, FUT175 was reported to prevent cerebral vasospasm,^69^ thrombosis in disseminated intravascular coagulation,^70^ and delayed ischemic neurological deficit in SAH.^71^ It could inhibit thrombin and preserve BBB integrity via the PKCα/RhoA/MLC2 pathway^72^ and promote axonal regeneration by activating the BDNF/TrkB/ERK1/2‐CREB pathway.^73^ C1‐INH has not been tested clinically in stroke patients yet, which has great potential in treating ischemic dysfunctions.

There were some limitations in our meta‐analysis. The overall analysis of clinical studies showed high heterogeneity, especially in IS studies, which were discussed above. Besides, we could not relate the change of serpin proteins in stroke patients to their clinical outcomes. Because most studies included did not report the severity of stroke, such as the NIHSS score. Therefore, we could only relate serpin proteins to the progression of IS, which showed consistency of serpin involvement after IS onset. In our meta‐analysis of preclinical studies, there were limited data included in many serpin groups, such as AT. In turn, some serpin proteins which researchers have focused on in the preclinical area have not been investigated clinically, such as C1‐INH. Therefore, we call for more communication between the clinical and preclinical branches and more attention to the function and utility of serpin family proteins in stroke progression, diagnosis, and treatment.

## 5 CONCLUSIONS

The decreasing AT level and increasing TAT level could be used as blood biomarkers (maybe in combination with other circulating factors) in the clinical diagnosis of acute and subacute IS. Preclinical evidence supported that C1‐INH and FUT175 effectively reduced the brain infarct size and improved animal behavior by rescuing motor and sensorimotor functions in MCAO models, which showed their potential as therapeutic drugs for IS.

## AUTHOR CONTRIBUTIONS

BY, L Luo, L Li, ZW, and RC performed literature search, screening, and risk of bias assessment, and conducted data extraction. BY performed data analysis and drafted the manuscript. XX and YW conducted the study conception and design, revised the manuscript, and supervised the project. All authors read and approved the final manuscript.

## FUNDING INFORMATION

This work was supported by the National Key R&D Program of China (2021ZD0202805 and 2019YFA0709504), the National Natural Science Foundation of China (31900719), the Innovative Research Team of High‐level Local Universities in Shanghai, Science and Technology Committee Rising‐Star Program (19QA1401400), 111 Project (B18015), Shanghai Municipal Science and Technology Major Project (2018SHZDZX01), and Shanghai Center for Brain Science and Brain‐Inspired Technology.

## CONFLICT OF INTEREST STATEMENT

No conflicts of interest were disclosed.

## Supporting information


Appendix S1

Appendix S2

## Data Availability

The data that support the findings of this study are available from the corresponding author upon reasonable request.
