# Supplementary material for: Serpin family proteins as potential biomarkers and therapeutic drugs in stroke: A systematic review and meta‐analysis on clinical/preclinical studies
Source: CNS Neurosci Ther. 2023 Apr 5;29(7):1738–49. doi: 10.1111/cns.14205 (PMC10324362; doi:10.1111/cns.14205)

Supplementary files

Supplementary 1. Search string (page 2-3)

Clinical studies:

Supplementary 2. Study characteristics of clinical studies (page 4-14)

Supplementary 3. Quality assessment of clinical studies (page 15-16)

Supplementary 4. Publication bias of clinical studies (page 17)

Supplementary 5. Sensitivity analysis of clinical studies (page 18)

Preclinical studies:

Supplementary 6. Study characteristics of preclinical studies (page 19-29)

Supplementary 7. Quality of preclinical studies (page 30-31)

Supplementary 8. Publication bias of preclinical studies (page 32)

Supplementary 9. Sensitivity analysis of preclinical studies (page 33)

Supplementary 1. Search string

Database 1. Web of science

#1 serpin

serpin OR "serine proteinase inhibitor" OR "serine protease inhibitor"

#2 stroke

stroke OR "cerebral ischemia" OR "brain ischemia" OR "intracranial hemorrhage" OR "cerebral hemorrhage"

Database 2. EMBASE (from OVID)

#1 serpin

serpin* OR serine proteinase inhibitor OR serine protease inhibitor

#2 stroke

stroke OR cerebral ischemia OR brain ischemia OR intracranial hemorrhage OR cerebral hemorrhage

Database 3. BIOSIS (from OVID)

#1 serpin

serpin* OR serine proteinase inhibitor OR serine protease inhibitor

#2 stroke

stroke OR cerebral ischemia OR brain ischemia OR intracranial hemorrhage OR cerebral hemorrhage

Database 4. MEDLINE (from OVID)

#1 serpin

serpin* OR serine proteinase inhibitor OR serine protease inhibitor

#2 stroke

stroke OR cerebral ischemia OR brain ischemia OR intracranial hemorrhage OR cerebral hemorrhage

Database 5. Pubmed

#1 serpin

"serpins"[MeSH Terms]

OR

"serpins"[tiab]

OR

"serpin" OR "serine proteinase inhibitor" OR "serine protease inhibitor"

#2 stroke

"stroke"[MeSH Terms] OR "MELAS Syndrome"[MeSH Terms] OR "brain ischemia"[MeSH Terms] OR "intracranial hemorrhages"[MeSH Terms]

OR

"stroke"[Tiab] OR "MELAS Syndrome"[Tiab] OR "brain ischemia"[Tiab] OR "intracranial hemorrhages"[Tiab]

OR

"stroke" OR "MELAS Syndrome" OR "brain ischemia" OR "intracranial hemorrhage" OR "cerebral ischemia" OR "cerebral hemorrhage"

Database 6. CNKI (Chinese database 知网)

Search strings in Simplified Chinese as below

丝氨酸蛋白酶抑制剂 AND 中风

Supplementary 2. Study characteristics of clinical studies

Summary

Note: gray shade means no study identified

Serpin	Synonyms	No. of studies	Types of stroke			No. of patients	No. of control
			IS	HS	TIA		
SERPINA1	a1-antitrypsin	5	Ischemic stroke, acute cerebrovascular ischemia	SAH	TIA	203	291
SERPINA3	a1-ACT	2	Cerebral Infarction	ICH		86	84
SERPINA12	Vaspin	3	Ischemic stroke	Hemorrhagic stroke		453	343
SERPINC1	AT	12	Ischemic stroke, cerebral ischemia, cerebral thrombosis, cardioembolic, atherothrombotic, lacunar	SAH, ICH	TIA	1053	569
SERPINC1-thrombin	TAT	15	Ischemic stroke, cerebral ischemia, cerebral thrombosis, cardioembolic, atherothrombotic, lacunar, large artery	SAH, ICH	TIA	1323	590
SERPINC1-FVIIa	FVIIa-AT	1	Ischemic stroke			33	20
SERPINI1	Neuroserpin	1	Ischemic stroke			133	44
SERPINE1	PAI-1	19	Ischemic stroke, cerebral ischemia, cerebral thrombosis, cardioembolic, atherothrombotic, lacunar, cerebral infaction, large artery, embolic, boundary, undertermined	SAH		1803	1307
SERPINB9P1	——	1	Ischemic stroke			159	153

Detailed study characteristics

No.	Author (year)	Serpin	Synonyms	Types of stroke	Subgroups	Stroke patients				Healthy control				Method
						Total number	male	female	age/year	Total number	male	female	age/year	
1	Gaetani (1996)	SERPINA1	a1-antitrypsin	HS	SAH	34	21	13	51±3	10	NA	NA	48±2	ELISA (serum)
2	Sakai (1999)	SERPINA1	a1-antitrypsin	HS	SAH	18	6	12	54±14	22	10	12	52±14	ELISA (plasma)
3	Tartara (1996)	SERPINA1	a1-antitrypsin	HS	SAH	20	NA	NA	NA	10	NA	NA	NA	ELISA (serum)
4	Burghaus (2006)	SERPINA1	a1-antitrypsin	IS	——	81	NA	NA	2.5 years (1 month to 18 years)	229	NA	NA	NA	ELISA (plasma)
5	Bartosik-Psujek (2003)	SERPINA1	a1-antitrypsin	IS, TIA	Acute cerebrovascular ischemia; TIA	30/20	18	12	62.3±6	20	10	10	60.3±5	ELISA (serum)
6	Syrjanen (1989)	SERPINA3	a1-ACT	IS	Cerebral Infarction	46	NA	NA	38(17-49)	46	NA	NA	NA	ELISA (plasma)
7	Shen (2021)	SERPINA3	a1-ACT	HS	ICH	40	25	15	64.6±7.8	38	24	14	64.0±10.1	ELISA (plasma)
8	Yu (2021)	SERPINA12	Vaspin	IS, HS	Ischemic stroke; Hemorrhagic stroke	156/79	NA	NA	58.43±10.75	235	NA	NA	57.16±13.38	ELISA (serum)
9	Cura (2014)	SERPINA12	Vaspin	IS	——	50	28	22	69.58±12.21	50	25	25	67.06±10.19	ELISA (serum)
10	Kadoglou (2014)	SERPINA12	Vaspin	IS	——	168	80	88	70±9	58	28	30	68±10	ELISA (plasma)
11	Lu (1989)	SERPINC1	AT	IS, HS, TIA	Ischemic stroke; ICH; TIA	43/4/28	55	20	59.6	27	18	9	53.7	Coagulation method (plasma)
12	Tohgi (1990)	SERPINC1	AT	IS	Cerebral thrombosis; Cerebral embolism	40/9	NA	NA	NA	69	28	41	61.0±8.6	ELISA
13	Haapaniemi (2002)	SERPINC1	AT	IS	——	55	40	15	60.2±11.4	55	NA	NA	60.0±11.4	ELISA (plasma)
14	Morena-Barrio (2015)	SERPINC1	AT	IS	——	117	39%	61%	77 (68–81)	97	32%	68%	41 (35–51)	ELISA (plasma)
15	Chen (2002)	SERPINC1	AT	IS	Cerebral ischemia (blood aβ GPI more than 6 U/ml; blood aβ GPI less than 6 U/ml)	16/95	71	40	61.3	30	NA	NA	NA	ELISA (plasma)

No.	Author (year)	Serpin	Synonyms	Types of stroke	Subgroups	Stroke patients				Healthy control				Method
						Total number	male	female	age/year	Total number	male	female	age/year	
16	Yamazaki (1993)	SERPINC1; TAT	AT; TAT	IS	Cardioembolic; Atherothrombotic; Lacunar	52/32/54 (in acute phase: 23/10/12)	32/17/44	20/15/10	63.7±18.5/63.4±12.2 /68.1±9.4	27 (27 as in result, but 23 in method)	11	12	60±12	ELISA (plasma)
17	Meng (2011)	SERPINC1; TAT	AT; TAT	IS	ischemic stroke	152	86	66	58.72±8.32	46	24	22	51.89±7.04	ELISA (plasma)
18	Kataoka (2000)	SERPINC1; TAT	AT; TAT	IS	Cardioembolic; Atherothrombotic; Lacunar	38/41/58	26/28/36	12/13/22	71.4/66.5/67.6	32	17	15	66.5	ELISA (plasma)
19	Seki (1995)	SERPINC1; TAT	AT; TAT	IS	acute-phase cerebral thrombosis (ACT); chronic-phase cerebral thrombosis(CCT); chronic-phase cerebra hemorrhage(CCH)	28/36/6	34	36	71±9	37	14	23	61±20	ELISA (plasma)
20	Ji (2014)	SERPINC1; SERPINE1; TAT	AT; PAI-1; TAT	HS	SAH	30	15	15	65.2±7.3	20	8	12	61.3±8.2	ELISA (blood)
21	Tohgi (1993)	SERPINC1; SERPINE1; TAT	AT; PAI-1; TAT	IS	Cerebral Thrombosis	33	NA	NA	NA	69	28	41	61.0±8.6	ELISA (serum)
22	Altes (1995)	SERPINC1; SERPINE1; TAT	AT; PAI-1; TAT	IS	Cardioembolic; Atherothrombotic; Lacunar	10/55/21	52	34	64±10.5	60	35	25	59±4.3	ELISA (serum)
23	Ye (2020)	TAT	TAT	IS	——	236	145	91	70 (62–79)	90	55	35	69	ELISA (plasma)
24	Ono (1991)	TAT	TAT	IS	Ischemic stroke (acute stroke and chronic stroke)	98(54/44)	56(30/26)	42(24/18)	69.5±13.3/69.6±10.9	50	32	18	66.0±11.0	ELISA (plasma)
25	Fon (1994)	TAT	TAT	TIA	TIA group; Remote TIA	36/20	28	28	68.3	65	23	42	65.5(51-81)	ELISA (plasma)
26	Ince (1999)	TAT	TAT	IS	——	32	16	16	30-80	21	9	12	45–73	ELISA (plasma)
27	Takano (1992)	TAT	TAT	IS	Cardioembolic; Atherothrombotic; Lacunar	54(21/10/23)	37	17	65.8	20	NA	NA	NA	ELISA (plasma)
28	Wu (2011)	TAT	TAT	HS	ICH (NIHSS(22-23); NIHSS(24-25); NIHSS(26-27))	13/30/17	36	24	60.33±12.79	10	NA	NA	NA	ELISA (plasma)
29	Johansson (2000)	SERPINE1	PAI-1	IS, HS	Infarction; Hemorrhage	87/18	62%	38%	55.1 (7.7)	216	62%	38%	55.1 (7.7)	ELISA (serum)
30	Margaglione (1994)	SERPINE1	PAI-1	IS	——	106	55	51	61.3	106	57	49	66.5	ELISA (plasma)

No.	Author (year)	Serpin	Synonyms	Types of stroke	Subgroups	Stroke patients				Healthy control				Method
						Total number	male	female	age/year	Total number	male	female	age/year	
31	Wang (2005)	SERPINE1	PAI-1	IS	Atherosclerotic ischemic stroke	60	32	28	68.6±11.8	60	32	28	67.2±11.2	ELISA (plasma)
32	Kain (2001)	SERPINE1	PAI-1	IS	Males and Females	90/50	90	50	65 (44±96)/67 (43±88)	38/52	38	52	59 (35±46)/60 (49±75)	ELISA (plasma)
33	Ilhan (2010)	SERPINE1	PAI-1	IS	Symptomatic lacunar stroke	30	19	11	65.40±10.8	30	16	14	59.96±8.3	ELISA (plasma)
34	Zhang (1995)	SERPINE1	PAI-1	IS	Cerebral Infarction (CACI/PACI)	62(30/32)	28(12/16)	34(18/16)	59.3±8.9/62.0±9.0	30	NA	NA	NA	ELISA (plasma)
35	Mansfield (1998)	SERPINE1	PAI-1	IS	——	80	48	32	72 (64–77)	80	48	32	68 (56–80)	ELISA (serum)
36	Vucković (2010)	SERPINE1	PAI-1	IS	——	60	39	21	63.5±9.6	30	18	12	60.2±8.0	ELISA (serum)
37	Macko (1996)	SERPINE1	PAI-1	IS	Stroke with infection inflammation; Stroke with no infection inflammation	18(9/9)	NA	NA	NA	17	NA	NA	NA	ELISA (plasma)
38	Ferro (1993)	SERPINE1	PAI-1	IS	Young Ischemic Stroke (lupus anticoagulant(LA)(+); lupus anticoagulant(LA)(-))	33(6/27)	27	6	23-49	25	NA	NA	NA	ELISA (plasma)
39	Margaglione (1996)	SERPINE1	PAI-1	IS	Large artery; Multiple (embolic); Lacunar; Boundary; Undetermined	101(48/20 /10/10/13)	51 (50.5)	50	66.2±10.0	109	57 (52.3)	52	61.2±11.9	ELISA (plasma)
40	Rooth (2011)	SERPINE1	PAI-1	IS	——	17 (17 in result, 20 in method)	13	7	74±13	22 (22 in result, 23 in method)	14	9	72±8	ELISA (plasma)
41	Zunker (1999)	SERPINE1	PAI-1	IS	——	89	55	34	65±11	19	11	8	69±8	ELISA (plasma)
42	Kubota (2021)	SERPINE1	PAI-1	IS	aCI, cCI, TIA	612 (459/65/88)	365	247	NA	281	183	98	NA	ELISA (serum)
43	Song (2006)	SERPINE1; TAT	PAI-1; TAT	IS	——	46	34	12	63.7±7.3	28	20	8	59.6±8.0	ELISA (plasma)
44	Topcuoglu (2000)	SERPINE1; TAT	PAI-1; TAT	IS	Lone AF; cardioembolism (CE); Atherothrombotic large artery disease (AL)	95 (21/24/50)	50	45	62.36	15	22	14	62.35	ELISA (plasma)
45	Wu (2017)	SERPINI1	Neuroserpin	IS	——	133	78	55	68.43±9.746	44	25	19	66.70±8.799	ELISA (serum)

No.	***Author (year)***	***Serpin***	***Synonyms***	***Types of stroke***	***Subgroups***	***Stroke patients***				***Healthy control***				***Method***
						Total number	***male***	***female***	***age/year***	***Total number***	***male***	***female***	***age/year***	
46	Słomka (2017)	FVIIa-AT	FVIIa-AT	IS	——	33	20	13	70 (65-80)	20	10	10	68 (42-86)	ELISA (plasma)
47	Huang (2021)	SERPINB9P1	SERPINB9P1	IS	——	159	97	62	66.35±11.10	153	71	82	65.49±7.14	qRT-PCR

Abbreviation (alphabetically)：

In serpins:

AT: antithrombin

α1-ACT: αl-antichymotrypsin

FVIIa-AT: factor VIIa–antithrombin complexes

In stroke types and subgroups:

aCI: acute cerebral infarction

AF: atrial fibrillation

CACI: cortical arterial cerebral infarction

cCI: chronic cerebral infarction

GPI: glycoprotein I

HS: hemorrhagic stroke

ICH: intracerebral hemorrhage

IS: ischemic stroke

PACI: perforating arterial cerebral infarction

SAH: subarachnoid hemorrhage

TIA: transient ischemic attack

47 included clinical studies

1. Gaetani P, Tartara F, Tancioni F, Klersy C, Forlino A, Baena RR: Activity of alpha 1-antitrypsin and cigarette smoking in subarachnoid haemorrhage from ruptured aneurysm. J Neurol Sci 1996, 141(1-2):33-38.

2. Sakai N, Nakayama K, Tanabe Y, Izumiya Y, Nishizawa S, Uemuara K: Absence of plasma protease-antiprotease imbalance in the formation of saccular cerebral aneurysms. Neurosurgery 1999, 45(1):34-38; discussion 38-39.

3. Tartara F, Gaetani P, Tancioni F, Guagliano A, Klersy C, Forlino A, Marzatico F, Rodriguez y Baena R: Alpha 1-antitrypsin activity in subarachnoid hemorrhage. Life Sci 1996, 59(1):15-20.

4. Burghaus B, Langer C, Thedieck S, Nowak-Göttl U: Elevated alpha1-antitrypsin is a risk factor for arterial ischemic stroke in childhood. Acta Haematol 2006, 115(3-4):186-191.

5. Bartosik-Psujek H, Belniak E, Stelmasiak Z: Markers of inflammation in cerebral ischemia. Neurol Sci 2003, 24(4):279-280.

6. Syrjänen J, Teppo AM, Valtonen VV, Iivanainen M, Maury CP: Acute phase response in cerebral infarction. J Clin Pathol 1989, 42(1):63-68.

7. Shen Y, Yang W, Xiong X, Li X, Xiao Z, Yu J, Liu F, Gui S, Xie X, Lv F et al: Integrated Multiomics Analysis Identifies a Novel Biomarker Associated with Prognosis in Intracerebral Hemorrhage. Oxid Med Cell Longev 2021, 2021:2510847.

8. Yu D, Huang B, Wu B, Xiao J: Association of serum vaspin, apelin, and visfatin levels and stroke risk in a Chinese case-control study. Medicine 2021, 100(12).

9. Cura HS, Ozdemir HH, Demir CF, Bulut S, Ilhan N, Inci MF: Investigation of Vaspin Level in Patients with Acute Ischemic Stroke. Journal of Stroke & Cerebrovascular Diseases 2014, 23(3):453-456.

10. Kadoglou NPE, Fotiadis G, Lambadiari V, Maratou E, Dimitriadis G, Liapis CD: Serum levels of novel adipokines in patients with acute ischemic stroke: Potential contribution to diagnosis and prognosis. Peptides 2014, 57:12-16.

11. Lu J: [Observation of plasma levels of antithrombin-III and plasminogen in acute cerebrovascular disease]. Zhonghua Shen Jing Jing Shen Ke Za Zhi 1989, 22(4):205-

207, 252-203.
12. Tohgi H, Kawashima M, Tamura K, Suzuki H: Coagulation-fibrinolysis abnormalities in acute and chronic phases of cerebral thrombosis and embolism. Stroke 1990, 21(12):1663-1667.
13. Haapaniemi E, Tatlisumak T, Soinne L, Syrjälä M, Kaste M: Natural anticoagulants (antithrombin III, protein C, and protein S) in patients with mild to moderate ischemic stroke. Acta Neurol Scand 2002, 105(2):107-114.
14. de la Morena-Barrio ME, Garcia A, Martinez-Martinez I, Minano A, Padilla J, Navarro-Fernandez J, Roldan V, Aguila S, Iniesta JA, Corral J et al: A new method to quantify beta-antithrombin glycoform in plasma reveals increased levels during the acute stroke event. Thrombosis Research 2015, 136(3):634-641.
15. Chen WH, Kao YF, Lan MY, Chang YY, Liu JS: A perturbation of antithrombin-III and protein C coupling associates with an increase of anti-beta2-glycoprotein I antibody in non-antiphospholipid antibody syndrome cerebral ischemia. Blood Coagul Fibrinolysis 2002, 13(8):703-709.
16. Yamazaki M, Uchiyama S, Maruyama S: Alterations of haemostatic markers in various subtypes and phases of stroke. Blood Coagul Fibrinolysis 1993, 4(5):707-712.
17. Meng R, Li ZY, Ji X, Ding Y, Meng S, Wang X: Antithrombin III associated with fibrinogen predicts the risk of cerebral ischemic stroke. Clin Neurol Neurosurg 2011, 113(5):380-386.
18. Kataoka S, Hirose G, Hori A, Shirakawa T, Saigan T: Activation of thrombosis and fibrinolysis following brain infarction. J Neurol Sci 2000, 181(1-2):82-88.
19. Seki Y, Takahashi H, Wada K, Shibata A: Sustained activation of blood coagulation in patients with cerebral thrombosis. Am J Hematol 1995, 50(3):155-160.
20. Ji Y, Meng QH, Wang ZG: Changes in the coagulation and fibrinolytic system of patients with subarachnoid hemorrhage. Neurol Med Chir (Tokyo) 2014, 54(6):457-464.
21. Tohgi H, Takahashi H, Chiba K, Tamura K: Coagulation-fibrinolysis system in poststroke patients receiving antiplatelet medication. Stroke 1993, 24(6):801-804.
22. Altès A, Abellán MT, Mateo J, Avila A, Martí-Vilalta JL, Fontcuberta J: Hemostatic

disturbances in acute ischemic stroke: a study of 86 patients. Acta Haematol 1995, 94(1):10-15.

23. Ye N, Liu Z, Wang X, Xu X, Wu W: Evaluation of analytic and clinical performance of thrombin-antithrombin complex and D-dimer assay in prognosis of acute ischemic stroke. Blood Coagul Fibrinolysis 2020, 31(5):303-309.

24. Ono N, Koyama T, Suehiro A, Oku K, Fujikake K, Kakishita E: Clinical significance of new coagulation and fibrinolytic markers in ischemic stroke patients. Stroke 1991, 22(11):1369-1373.

25. Fon EA, Mackey A, Côté R, Wolfson C, McIlraith DM, Leclerc J, Bourque F: Hemostatic markers in acute transient ischemic attacks. Stroke 1994, 25(2):282-286.

26. Ince B, Bayram C, Harmanci H, Ulutin T: Hemostatic markers in ischemic stroke of undetermined etiology. Thromb Res 1999, 96(3):169-174.

27. Takano K, Yamaguchi T, Uchida K: Markers of a hypercoagulable state following acute ischemic stroke. Stroke 1992, 23(2):194-198.

28. Wu CH, Yang RL, Huang SY, Li HZ, Wang KY, Yang DH, Yan XH, Xue XH, Wu SY, Wang JM et al: Analysis of thrombin-antithrombin complex contents in plasma and hematoma fluid of hypertensive intracerebral hemorrhage patients after clot removal. Eur J Neurol 2011, 18(8):1060-1066.

29. Johansson L, Jansson JH, Boman K, Nilsson TK, Stegmayr B, Hallmans G: Tissue plasminogen activator, plasminogen activator inhibitor-1, and tissue plasminogen activator/plasminogen activator inhibitor-1 complex as risk factors for the development of a first stroke. Stroke 2000, 31(1):26-32.

30. Margaglione M, Di Minno G, Grandone E, Vecchione G, Celentano E, Cappucci G, Grilli M, Simone P, Panico S, Mancini M: Abnormally high circulation levels of tissue plasminogen activator and plasminogen activator inhibitor-1 in patients with a history of ischemic stroke. Arterioscler Thromb 1994, 14(11):1741-1745.

31. Wang J, Li J, Liu Q: Association between platelet activation and fibrinolysis in acute stroke patients. Neurosci Lett 2005, 384(3):305-309.

32. Kain K, Catto AJ, Carter AM, Young J, Bamford J, Bavington J, Grant PJ: Decreased fibrinolytic potential in South Asian women with ischaemic cerebrovascular

disease. Br J Haematol 2001, 114(1):155-161.
33. Ilhan D, Ozbabalik D, Gulcan E, Ozdemir O, Gulbacs Z: Evaluation of Platelet Activation, Coagulation, and Fibrinolytic Activation in Patients With Symptomatic Lacunar Stroke. Neurologist 2010, 16(3):188-191.
34. Zhang Y, Liu X, Cai Z: [An analysis on the activity status of fibrinolytic system in Chinese patients with acute cerebral infarction]. Zhonghua Nei Ke Za Zhi 1995, 34(8):518-521.
35. Mansfield MW, Catto AJ, Carter AM, Grant PJ: Fibrinolytic measurements in type 2 diabetic patients with acute cerebral infarction. Diabet Med 1998, 15(11):953-957.
36. Vucković BA, Djerić MJ, Ilić TA, Canak VB, Kojić-Damjanov S, Zarkov MG, Cabarkapa VS: Fibrinolytic parameters, lipid status and lipoprotein(a) in ischemic stroke patients. Srp Arh Celok Lek 2010, 138 Suppl 1:12-17.
37. Macko RF, Ameriso SF, Gruber A, Griffin JH, Fernandez JA, Barndt R, Quismorio FP, Jr., Weiner JM, Fisher M: Impairments of the protein C system and fibrinolysis in infection-associated stroke. Stroke 1996, 27(11):2005-2011.
38. Ferro D, Quintarelli C, Rasura M, Antonini G, Violi F: Lupus anticoagulant and the fibrinolytic system in young patients with stroke. Stroke 1993, 24(3):368-370.
39. Margaglione M, DiMinno G, Grandone E, Celentano E, Vecchione G, Cappucci G, Grilli M, Mancini FP, Postiglione A, Panico S et al: Plasma lipoprotein(a) levels in subjects attending a metabolic ward. Discrimination between individuals with and without a history of ischemic stroke. Arterioscler Thromb Vasc Biol 1996, 16(1):120-128.
40. Rooth E, Wallen NH, Blomback M, He S: Decreased fibrin network permeability and impaired fibrinolysis in the acute and convalescent phase of ischemic stroke. Thrombosis Research 2011, 127(1):51-56.
41. Zunker P, Schick A, Padró T, Kienast J, Phillips A, Ringelstein EB: Tissue plasminogen activator and plasminogen activator inhibitor in patients with acute ischemic stroke: relation to stroke etiology. Neurol Res 1999, 21(8):727-732.
42. Kubota M, Yoshida Y, Kobayashi E, Matsutani T, Li SY, Zhang BS, Mine S, Machida T, Takizawa H, Hiwasa T et al: Serum anti-SERPINE1 antibody as a potential

biomarker of acute cerebral infarction. Sci Rep 2021, 11(1):21772.
43. Song JW, Song KS, Choi JR, Kim SY, Rhee JH: Plasma level of IL-6 and its relationship to procoagulant and fibrinolytic markers in acute ischemic stroke. Yonsei Med J 2006, 47(2):201-206.
44. Topcuoglu MA, Haydari D, Ozturk S, Ozcebe OI, Saribas O: Plasma levels of coagulation and fibrinolysis markers in acute ischemic stroke patients with lone atrial fibrillation. Neurol Sci 2000, 21(4):235-240.
45. Wu W, Asakawa T, Yang Q, Zhao J, Lu L, Luo Y, Gong P, Han S, Li W, Namba H et al: Effects of neuroserpin on clinical outcomes and inflammatory markers in Chinese patients with acute ischemic stroke. Neurological Research 2017, 39(10):862-868.
46. Słomka A, Świtońska M, Sinkiewicz W, Żekanowska E: Assessing Circulating Factor VIIa-Antithrombin Complexes in Acute Ischemic Stroke: A Pilot Study. Clin Appl Thromb Hemost 2017, 23(4):351-359.
47. Huang J, Zhu L, Zhao X, Wu X, Yang J, Xu B, Zhao Z, Gu L, Su L: LncRNA SERPINB9P1 expression and polymorphisms are associated with ischemic stroke in a Chinese Han population. Neurol Sci 2022, 43(2):1143-1154.

Supplementary 3. Quality assessment of clinical studies

Risk of bias assessment

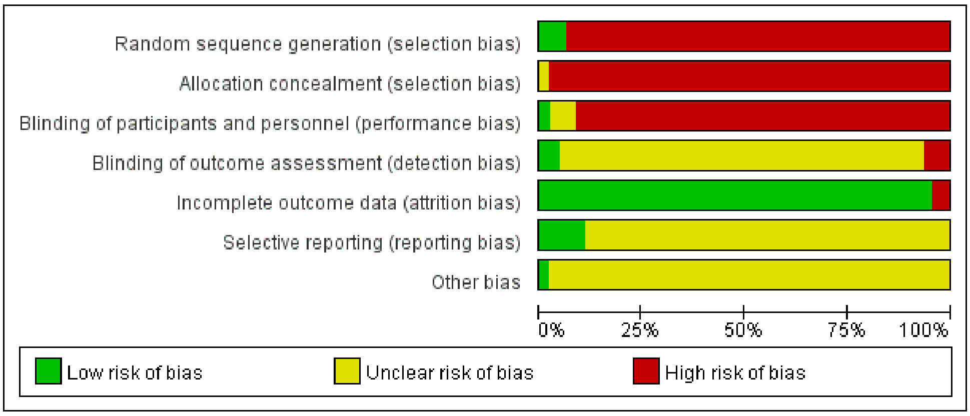

Risk of bias of 47 studies included

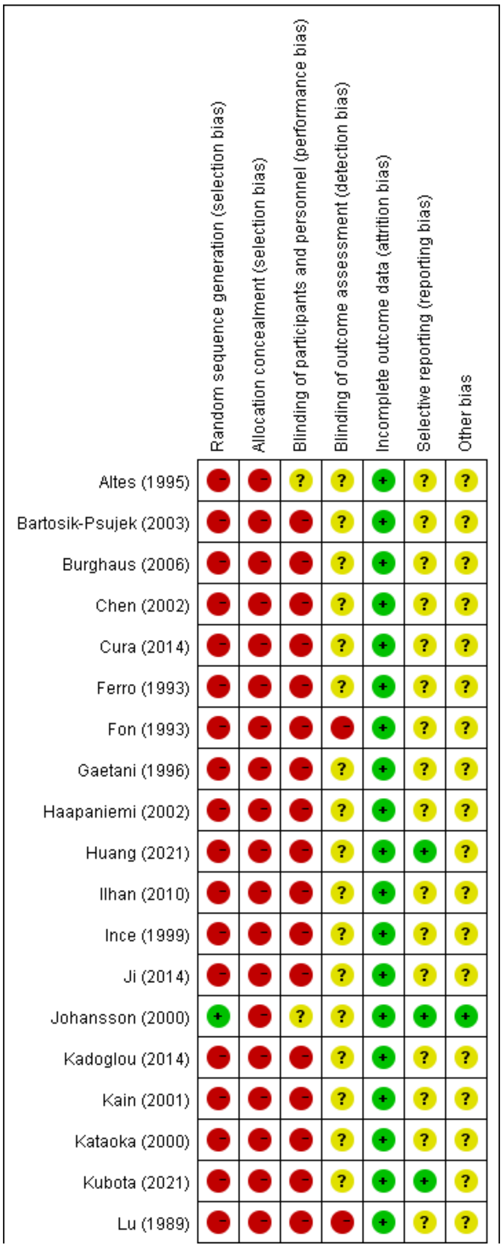

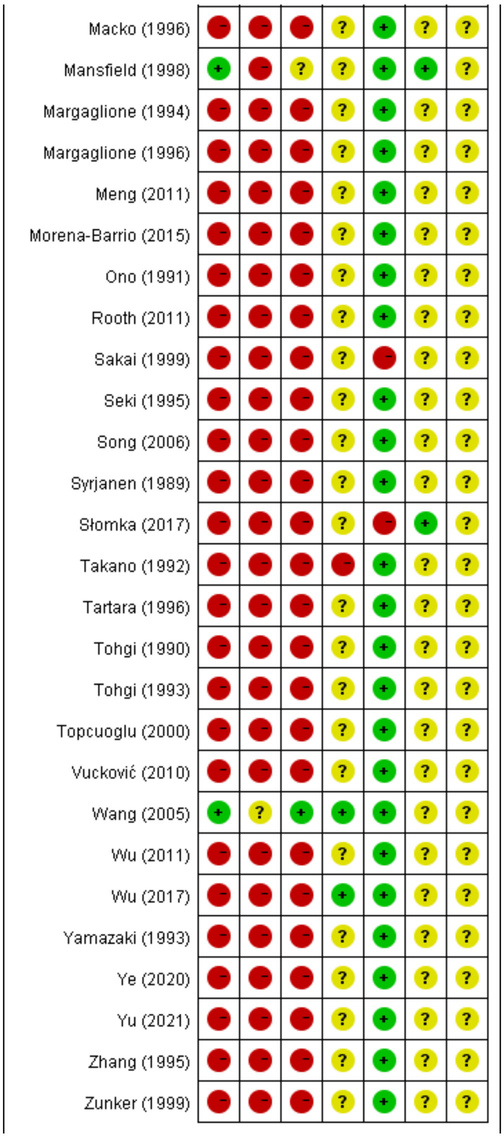
Macko (1996)
Mansfield (1998)
Margaglione (1994)
Margaglione (1996)
Meng (2011)
Morena-Barrio (2015)
Ono (1991)
Rooth (2011)
Sakai (1999)
Seki (1995)
Song (2006)
Syrjanen (1989)
Słomka (2017)
Takano (1992)
Tartara (1996)
Tohgi (1990)
Tohgi (1993)
Topcuoglu (2000)
Vucković (2010)
Wang (2005)
Wu (2011)
Wu (2017)
Yamazaki (1993)
Ye (2020)
Yu (2021)
Zhang (1995)
Zunker (1999)

Supplementary 4. Publication bias of clinical studies

As shown by the asymmetry of funnel plots, there was publication bias in the studies included.

Each blue dot indicated an experiment. Red line indicated the overall effect size. Grey line showed 95%CI. The asymmetry of funnel plot suggested publication bias.

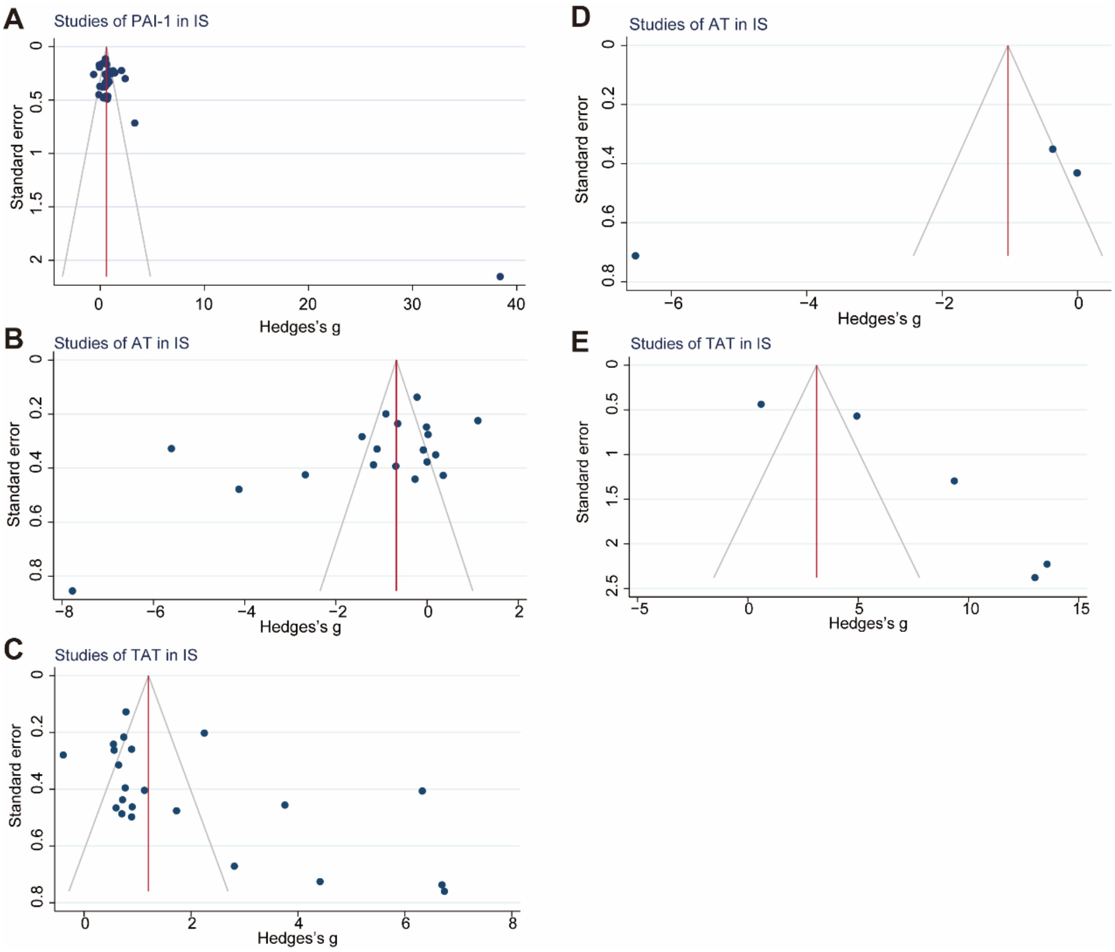

Supplementary 5. Sensitivity analysis of clinical studies

We used leave-one-out method to assess the influence of single study on the overall effect and the robustness of meta-analysis. One study was omitted and meta-analysis was performed on the remaining studies. The Hedges's g of leave-one-out analysis fell into the 95% CI of the orginal effect size.

Study ID indicated the removed study. Red lines indicated the overall effect of all studies included and the 95%CI.

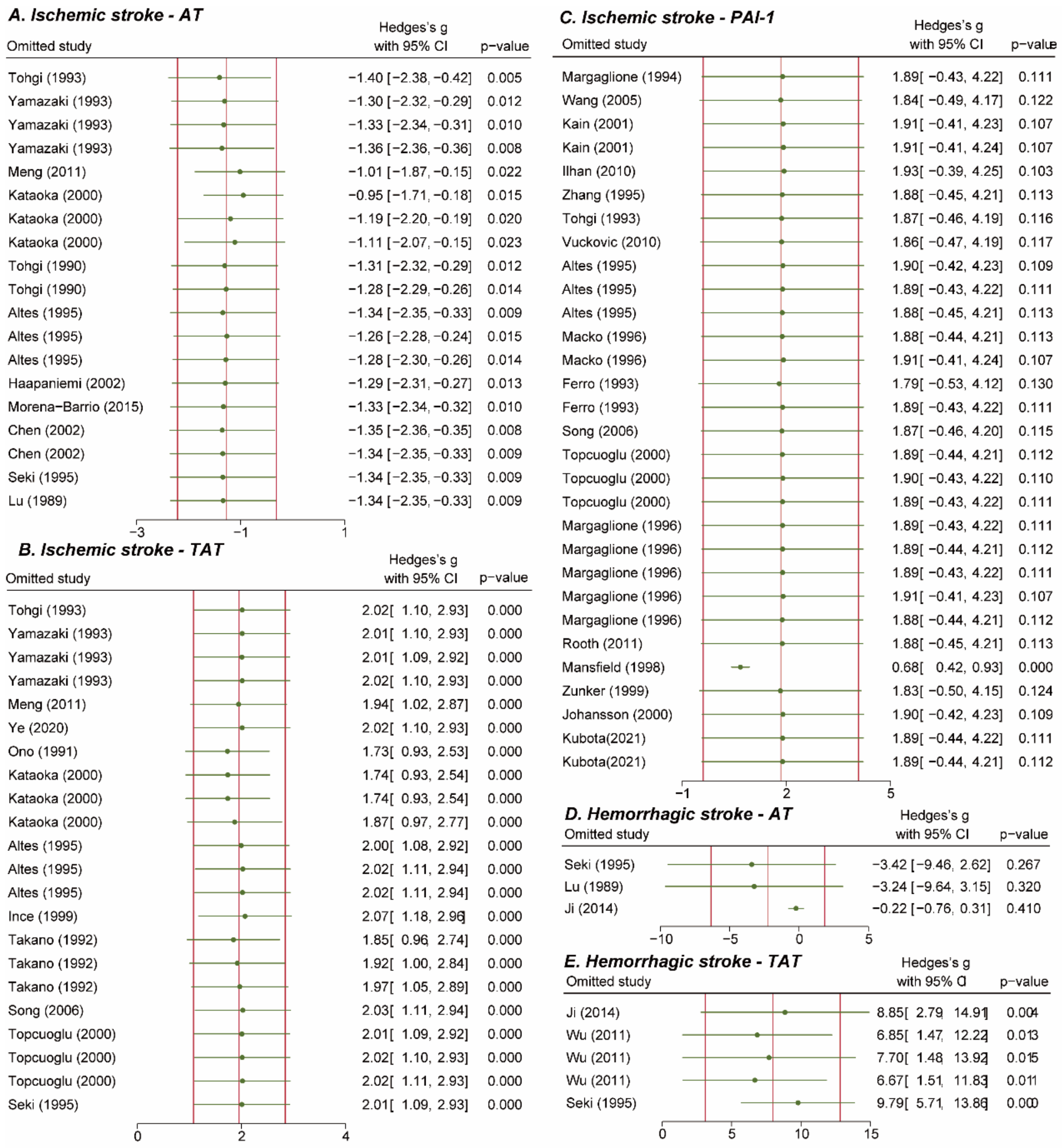

Supplementary 6. Study characteristics of preclinical studies

Summary

Note: gray shade means no study identified

Serpins		α1-Antitrypsin (SERPINA1)	LEX032 (SERPINA3)	α1-ACT (SERPINA3N)	PAI-2 (SERPINB2)	AT (SERPINC1)	PAI-1 (SERPINE1)	PEDF (SERPINF1)	C1-INH (SERPING1)	Neuroserpin (SERPINI1)	CrmA (SERPINN)	FUT175 (Artifact)	Aprotinin (Artifact)
**Number of Studies**		1 (2.4%)	1 (2.4%)	1 (2.4%)	1 (2.4%)	2 (5%)	6 (15%)	5 (12%)	10 (24%)	4 (10%)	1 (2.4%)	7 (17%)	2 (5%)
**Species**	*Rats*	1	1			1	4	3	4	1	1	5	2
	Mice			1	1	1	1	2	7	3			
	Others					Rabbits (1)	Pigs (1)					Rabbits (2)	
**Model**	*tMCAO*	1	1	1	1	1	1	5	8	2	1	5	1
	pMCAO					1	1		2				
	Photothrombosis						1		1				
	Others					SAH (1)	HI (4)		Thromboembolic MCAO (1)	IR (1)<br>ICH (1)		SAH (2)	IR (1)
**Injection**	*Intravenous*	1	1				2	2	8			7	2
	Intracranial	1		1						3	1		
	Intracerebro -ventricular				1		3	3					
	Others				Intranasal (1)	Intraperitoneal (2)<br>Intracisternal (1)	Intranasal (1)		Intraarterial (2)	Intravitreal (1)			
**Outcome**	*Infarct Size*	1	1	1	1	2	4	3	9	2		5	1
	Behavior Scores/Tests	1	1	1		2	2	1	7	1		5	1

Detailed study characteristics *(*indicated studies not included in meta-analysis)*

Note: For sourse information, see pharma company name below serpin name in column 'synonyms' and code number of the study in column 'auther (year)' when available.

No	author (year)	serpin	synonyms	model	species	sex	age	weight	No. of treatment group	control	No. of control group	dose	Injection method	Injection timepoint	outcome timepoint	neurological outcomes	behavioral outcomes
1	Moldthan (2014)	SERPINA1	α1-Antitrypsin (Grifols, USA)	tMCAO	rats	male	7-8 weeks	220-250g	3	PBS	3	70ug	intracranial	5-10min following ET-1 injection	POD3	infarct size	cylinder test<br>vibrissae test
1	Moldthan (2014)	SERPINA1	α1-Antitrypsin (Grifols, USA)	tMCAO	rats	male	7-8 weeks	220-250g	14	saline	8	40mg/kg	intravenous	5-10min following ET-1 injection	POD3	infarct size	cylinder test<br>vibrissae test
2	Weaver (2002)	SERPINA3	LEX032	tMCAO	rats	NA	NA	225-250g	5~6	vehicle	5~6	50mg/kg	intravenous	1 min prior to removal of suture from MCA	POD1	infarct size<br>brain swelling	neurological score
3	Zhang (2022)	SERPINA3N	α1-ACT (Genomeditech, China)	tMCAO	mice	male	8-12 weeks	NA	6	AAV-null-ZsGreen	6	2 × 10^9 genome copies	intracranial	4 weeks prior to MCAO	24h after surgery	infarct volume	Bederson's test
4	Buchthal (2018)	SERPINB2	PAI-2 (OriGene, Germany)	pMCAO	mice	male	8 weeks ± 5days	24-26g	16	aCSF	22	100ng	intracerebroventricular	within 10min after MCAO	POD7	infarct volume	NA
4	Buchthal (2018)	SERPINB2	PAI-2 (OriGene, Germany)	pMCAO	mice	male	8 weeks ± 5days	24-26g	10min: 10<br>60min: 6	GFP	10min: 12<br>60min: 6	1ug	intranasal	10min/60min, repeated 24 and 48h later	POD7	infarct volume	NA
5	Vollmer (1989) *	SERPINC1	AT	SAH	rabbits	male	NA	3-4kg	7	no injection	7	100U	intracisternal	POD3	POD3 immediately after drug injection	basilar artery diameters	NA
6	Cuomo (2007)	SERPINC1	AT	tMCAO	mice	male	NA	25-27g	7	vehicle	7	10IU/kg<br>30IU/kg	intraperitoneal	3h/3+6h/6h	POD1	infarct volume	general neurological score<br>focal neurological score
6	Cuomo (2007)	SERPINC1	AT	pMCAO	rats	male	NA	250-270g	6	vehicle	5	10IU/kg<br>30IU/kg	intraperitoneal	3h/3+6h/6h	POD1<br>/POD7	infarct volume	general neurological score<br>focal neurological score
7	Heimann (1999)	SERPING1	C1-INH (CSL Behring, Germany)	Photo-thrombosis	rats	male	NA	31&±44g	8	saline	8	20IU/kg	intraarterial	immediately after occlusion	POD5	Regional cerebral blood flow<br>infarct volume	NA
8	De Simoni (2004) RBAU01RXS2	SERPING1	C1-INH (Baxter-Immuno, Italy)	tMCAO	mice	male	NA	26-28g	6~9	saline	6~9	1/5/15U	intravenous	at the beginning of sichemia,<br>at reperfusion	POD1	infarct volume	general deficit score,<br>focal deficit score
9	Heydenreich (2012)	SERPING1	C1-INH (CSL Behring, Germany)	tMCAO	mice	male/ female	6 weeks /6 months /12 months	NA	varied	saline	varied	7.5/15 U	intravenous	1/6h post	POD1	infarct size(POD1/7), BBB, edema	global neurological function (Bederson score,POD1/5), motor function (grip test)
9	Heydenreich (2012)	SERPING1	C1-INH (CSL Behring, Germany)	pMCAO	mice	male	NA	NA	varied	saline	varied	7.5/15 U	intravenous	1h post	POD1	infarct size	global neurological function (Bederson score),<br>motor function (grip test)
9	Heydenreich (2012)	SERPING1	C1-INH (CSL Behring, Germany)	tMCAO	rats	male	NA	NA	varied	saline	varied	20U/kg	intravenous	90min post	POD1	infarct size, edema	global neurological function (Bederson score),<br>motor function (grip test)

No	author (year)	serpin	synonyms	model	species	sex	age	weight	No. of treatment group	control	No. of control group	dose	Injection method	Injection timepoint	outcome timepoint	neurological outcomes	behavioral outcomes
10	Storini (2004) * RBAU01RXS2	SERPING1	C1-INH (Baxter-Immuno, Italy)	tMCAO	mice	male	NA	26-28g	varied	saline	varied	15U	intravenous	beginning of the ischemia	POD2	NA	general/focal deficit
11	Tomasi (2011)	SERPING1	C1-INH (Pharming, the Netherlands)	PartI: pMCAO+ICH PartII: thromboembolic MCAO	rats	male	NA	250-350g	4	saline	5	180IU/kg	intra-arterial	2h post occlusion	POD1	infarct size edema volume ICH visual score	neuroscore (2h/24h)
12	Akita (2003)	SERPING1	C1-INH	tMCAO	rats	male	10-13weeks	300-370g	19	saline	17	50IU/kg	intravenous	just befoe reperfusion	POD2	infarct size	NA
13	Chen (2018)	SERPING1	C1-INH (CSL Behring, Germany)	tMCAO	mice	male/ female	3 months; 13-14 months	NA	varied	saline	varied	75/150/300 U/kg	intravenous	30min pre, 1h/3h/6h post	POD3	infarct size	neurological score (24/48/72h)
14	De Simoni (2003)	SERPING1	C1-INH (Baxter-Immuno, Italy)	tMCAO	mice	male	NA	26-28g	varied	saline	varied	15U	intravenous	5min after onset of ischemia	POD2	infarct size	general/focal deficit
15	Mercurio (2021)	SERPING1	pdC1-INH /rhC1-INH (Pharming)	tMCAO	mice	male	9-11 weeks	20-30g	6	saline	6	15U	intravenous	at reperfusion	POD2	infarct size	neuroscore
16	Gesuete (2009)	SERPING1	pdC1-INH (Baxter-Immuno)	tMCAO	mice	male	NA	26-28g	varied	saline	varied	5/10/15U	intravenous	at the beginning of ischemia /3/6/18/24h post	NA	infarct size	NA
16	Gesuete (2009)	SERPING1	rhC1-INH (Pharming)	pMCAO	mice	male	NA	26-28g	varied	saline	varied	15U	intravenous	at the beginning of ischemia /3/6/18h post	NA	infarct size	NA
17	Gu (2015) *	SERPINI1	Neuroserpin (PeproTech, USA)	acute retinal IR injury	mice	male	8-12 weeks	16-20g	3	bovine serum albumin	3	1ul, 20umol/L	Intravitreal	at IR injury	POD1	NA	NA
18	Li (2017) *	SERPINI1	Neuroserpin (PeproTech, USA)	ICH	mice	male	8-12 weeks	25-35g	12	saline	12	not mentioned	intracranial	after ICH	same day as treatment	cerebral water content EB leakage	neurological score
19	Wu (2010)	SERPINI1	Neuroserpin	MCAO	mice	male	8-12 weeks	NA	12	PBS	12	3ul, 20-umol/L	intracranial	immediately after MCAO	POD1	Infarct size	NA
20	Yepes (2000)	SERPINI1	Neuroserpin	MCAO	rats	male	NA	350-400g	8	PBS	8	20ul, 30umol/L	intracranial	immediately after MCAO	POD3	infarct size	NA
21	Armstead (2014) *	SERPINE1	PAI-1 (EEIIMD)	HI	pigs	male/ female	1-5 day	1-1.4 kg	5	saline	5	1mg/kg	intravenous	30min before/ 60min after H/I	2h post H/I	pial artery diameter	NA
22	Yang (2013a)	SERPINE1	PAI-1 (Molecular Innovation)	LPS/HI	rats	NA	7 days	NA	4	PBS	4	1.9ug	intracerebroventricular	10 min/2h/4h post-hypoxia	POD1	brain damage(tissue loss), BBB leakage(NaF)	motor functions (30 or 42 days of age)

No	author (year)	serpin	synonyms	model	species	sex	age	weight	No. of treatment group	control	No. of control group	dose	Injection method	Injection timepoint	outcome timepoint	neurological outcomes	behavioral outcomes
23	Yang (2013b)	SERPINE1	PAI-1 (Molecular Innovation)	HI	rats	male/ female	7 days/30 days	NA	varied	PBS	varied	2ul, 0.24ug/ul	intranasal	30/120min after HI	POD1	brain damage(tissue loss), BBB leakage(NaF)	NA
24	Yang (2009)	SERPINE1	PAI-1 (Molecular Innovation)	HI	rats	NA	7 days	NA	varied	saline	varied	1.9ug, 0.95/1.9/2.85 /3.8ug	intracerebroventricular	at the end of HI, 1/2/4h post	4h/24h/7days post H	BBB leakage(Evans blue), edema(T2), MRI (ADC, FA)	NA
25	Nagai (2005)	SERPINE1	PAI-1	permanent MCA ligation/Photochemically induced thrombosis	mice	male	NA	20-30g	6	solvent	6	5ul, 0.38mg/ml	intracerebroventricular	after MCAO	POD1	infarct size	NA
26	Krakovsky (2011)	SERPINE1	PAI-1 (Novetide, Israel)	tMCAO	rats	male	3 monts	300-350g	18	vehicle	16	1mg/kg	intravenous	at the time of reperfusion	POD3	infarct size, edema	neurological score
27	Huang (2018)	SERPINF1	PEDF (GenePharma, China)	MCAO	rats	male	NA	230-280g	5	no treatment	5	100ug/kg/day for 3 days	intracerebroventricular	3 days before MCAO	POD3	infarct size	NA
28	Michalski (2017) *	SERPINF1	PEDF (Creative Biomart, USA)	MCAO	rats	male	NA	250-300g	14	saline	17	50mg/300g body weight	intravenously	3h after reperfusion (4h after ischemia onset)	POD7	NA	NA
29	Riabinska (2020)	SERPINF1	PEDF (Bioproducts MD, USA)	MCAO	mice	male	8-11 weeks	NA	7	CSF	7	36ul, 20ug/ml	intraventricular	48h prior to MCAO	POD1	lesion volume, edema, BBB integrity	NA
30	Zille (2014)	SERPINF1	PEDF	MCAO	mice	male	8-11 weeks	NA	5	CSF	5	84ul, 20ug/ml	intraventricular	48h prior to MCAO	POD1	infarct size, edema	open field (POD7), pole test (POD9), Rotarod (POD10), Morris water maze (POD21)
31	Pillai (2013) *	SERPINF1	PEDF (Creative Biomart, USA)	tMCAO	rats	male	NA	250-300g	14	saline	17	50ug/300g body weight	intravenous	3h after reperfusion (4h after ischemia onset)	POD1/2/4/7	edema, BBB integrity, vasculature	NA
32	Kwon (2015)	artifact	FUT175 (Sigma, USA)	tMCAO	rats	male	9 weeks	250-280g	NA	no treatme	NA	1mg/kg	intravenous	30min prior to and 30min after surgery	POD1	infarct size, edema	neurological score
33	Wang (2016)	artifact	FUT175 (Nanjing D&R Pharma,China)	tMCAO	rats	male	NA	250-280g	10-12	glucose	10-12	0.01/0.1/1mg/kg	intravenous	multiple: 0,2,4,6h after MCAO	POD1	infarct size, edema, BBB leakage	neurological score
34	Chen (2015)	artifact	FUT175 (Nanjing D&R Pharma,China)	tMCAO	rats	male	NA	260-280g	8-10	vehicle	8-10	0.01/0.1/0.3/1/3 mg/kg	intravenous	multiple: 0,2,4,6h after MCAO	POD1	infarct size, edema, BBB leakage	neurological score
35	Li (2016)	artifact	FUT175 (Nanjing D&R Pharma,China)	tMCAO	rats	male	NA	260-280g	11/9/10	glucose	12	0.01/0.1/1mg/kg	intravenous	multiple: 0,2,4,6h after MCAO	varied	infarct size [POD7]	corner test, grip-traction test, beam balance test, linb-placing test, longa test
36	Liu (2017)	artifact	FUT175 (Nanjing D&R Pharma,China)	tMCAO	rats	male	NA	250-300g	varied	vehicle	varied	0.01/0.1/1mg/kg	intravenous	multiple: 0,2,4,6h after MCAO	varied	infarct size [POD7]	corner test, grip-traction test, beam balance test, linb-placing test, ladder rung

No	author (year)	serpin	synonyms	model	species	sex	age	weight	No. of treatment group	control	No. of control group	dose	Injection method	Injection timepoint	outcome timepoint	neurological outcomes	behavioral outcomes
37	Yanamoto (1994) *	artifact	FUT175 (Torii Pharma, Japan)	SAH + latex beads-derived arterial narrowing	rabbits	male	NA	3-3.5kg	varied	no treatme	varied	3 x 2mg	intravenous	20min/12h/24h after beads	POD2	arterial caliber	NA
38	Yanamoto (1992) *	artifact	FUT175 (Torii Pharma, Japan)	SAH	rabbits	male	NA	3-3.5kg	varied	no treatme	10	3 x 1/2/3mg	intravenous	20min/12h/24h after SAH or POD2	POD1/2/3/4/7 OR 0/20/40/60min after injection	arterial caliber	NA
39	Eser (2007) *	artifact	Aprotinin (Bayer, Germany)	I/R	rats	male	NA	250-300g	6	no injectio	6	30000KIU/kg /0.7ml	intravenous	5min before reperfusion, contained at 10000KIU/kg/h/0.2ml during reperfusion	end of reperfusion	NA	NA
40	Homi (2010)	artifact	Aprotinin	MCAO+ CBP	rats	male	12-14weeks	275-300g	8	saline	8	60000kIU/kg loading dose, continuous infusion	intravenous	10min before MCAO	end of CPB, 2h, 24h POD3	infarct volume	neurological score
41	Sung (2007)	SERPINN (viral serpin)	CrmA	pMCAO + bilateral CCA occlusion	rats	male	NA	280-320g	12	control vec	12	5.5x10^6 to 7.5x10^6 particles/ml, 2ul	intracranial	12-16h before MCAO	POD2	X-gal staining and cell counts	NA

Abbreviations (alphabetically):

In serpins:

AT: Antithrombin

α1-ACT: α1-antichymotrypsin

C1-INH: C1 esterase inhibitor

PAI: plasminogen activator inhibitor

In stroke types/models:

CBP: cardiopulmonary bypass

CCA: common carotid artery

HI: hypoxic–ischemic

ICH: intracerebral hemorrhage

I/R: ischemia-reperfusion

pMCAO: permanent middle cerebral artery occlusion

SAH: subarachnoid hemorrhage

tMCAO: transient middle cerebral artery occlusion

In treatment and outcomes:

BBB: blood–brain barrier

CSF: cerebrospinal fluid (artificial)

EB: Evans blue (dye)

PBS :phosphate-buffered saline

POD: postoperatsive day

X-gal: 5'-Bromo-4-chloro-3-indoly-β-D-galactopyranoside

MRI: magnetic resonance imaging

NaF: sodium fluoride

ADC: apparent diffusion coefficient

FA: fractional anisotropy

41 included preclinical studies

1. Moldthan HL, Hirko AC, Thinschmidt JS, Grant MB, Li Z, Peris J, Lu Y, Elshikha AS, King MA, Hughes JA et al: Alpha 1-Antitrypsin Therapy Mitigated Ischemic Stroke Damage in Rats. Journal of Stroke & Cerebrovascular Diseases 2014, 23(5):E355-E363.

2. Weaver M, Leshley K, Sands H, Gritman KR, Legos JJ, Tuma RF: LEX032, a novel recombinant serpin, protects the brain after transient focal ischemia. Microvascular Research 2002, 63(3):327-334.

3. Zhang Y, Chen Q, Chen D, Zhao W, Wang H, Yang M, Xiang Z, Yuan H: SerpinA3N attenuates ischemic stroke injury by reducing apoptosis and neuroinflammation. CNS Neurosci Ther 2022, 28(4):566-579.

4. Buchthal B, Weiss U, Bading H: Post-injury Nose-to-Brain Delivery of Activin A and SerpinB2 Reduces Brain Damage in a Mouse Stroke Model. Molecular Therapy 2018, 26(10):2357-2365.

5. Vollmer DG, Hongo K, Kassell NF, Ogawa H, Tsukahara T, Lehman RM: Effect of intracisternal antithrombin III on subarachnoid hemorrhage-induced arterial narrowing. J Neurosurg 1989, 70(4):599-604.

6. Cuomo O, Pignataro G, Gala R, Scorziello A, Gravino E, Piazza O, Tufano R, Di Renzo G, Annunziato L: Antithrombin reduces ischemic volume, ameliorates neurologic deficits, and prolongs animal survival in both transient and permanent focal ischemia. Stroke 2007, 38(12):3272-3279.

7. Heimann A, Takeshima T, Horstick G, Kempski O: C1-esterase inhibitor reduces infarct volume after cortical vein occlusion. Brain Res 1999, 838(1-2):210-213.

8. De Simoni MG, Rossi E, Storini C, Pizzimenti S, Echart C, Bergamaschini L: The powerful neuroprotective action of C1-inhibitor on brain ischemia-reperfusion injury does not require C1q. Am J Pathol 2004, 164(5):1857-1863.

9. Heydenreich N, Nolte MW, Göb E, Langhauser F, Hofmeister M, Kraft P, Albert-Weissenberger C, Brede M, Varallyay C, Göbel K et al: C1-inhibitor protects from brain ischemia-reperfusion injury by combined antiinflammatory and antithrombotic mechanisms. Stroke 2012, 43(9):2457-2467.

10. Storini C, Rossi E, Marrella V, Distaso M, Veerhuis R, Vergani C, Bergamaschini L, De Simoni MG: C1-inhibitor protects against brain ischemia-reperfusion injury via inhibition of cell recruitment and inflammation. Neurobiol Dis 2005, 19(1-2):10-17.
11. Tomasi S, Sarmientos P, Giorda G, Gurewich V, Vercelli A: Mutant prourokinase with adjunctive C1-inhibitor is an effective and safer alternative to tPA in rat stroke. PLoS One 2011, 6(7):e21999.
12. Akita N, Nakase H, Kaido T, Kanemoto Y, Sakaki T: Protective effect of C1 esterase inhibitor on reperfusion injury in the rat middle cerebral artery occlusion model. Neurosurgery 2003, 52(2):395-400; discussion 400-391.
13. Chen X, Arumugam TV, Cheng YL, Lee JH, Chigurupati S, Mattson MP, Basta M: Combination Therapy with Low-Dose IVIG and a C1-esterase Inhibitor Ameliorates Brain Damage and Functional Deficits in Experimental Ischemic Stroke. Neuromolecular Med 2018, 20(1):63-72.
14. De Simoni MG, Storini C, Barba M, Catapano L, Arabia AM, Rossi E, Bergamaschini L: Neuroprotection by complement (C1) inhibitor in mouse transient brain ischemia. J Cereb Blood Flow Metab 2003, 23(2):232-239.
15. Mercurio D, Piotti A, Valente A, Oggioni M, Ponstein Y, Van Amersfoort E, Gobbi M, Fumagalli S, De Simoni MG: Plasma-derived and recombinant C1 esterase inhibitor: Binding profiles and neuroprotective properties in brain ischemia/reperfusion injury. Brain Behav Immun 2021, 93:299-311.
16. Gesuete R, Storini C, Fantin A, Stravalaci M, Zanier ER, Orsini F, Vietsch H, Mannesse ML, Ziere B, Gobbi M et al: Recombinant C1 inhibitor in brain ischemic injury. Ann Neurol 2009, 66(3):332-342.
17. Gu RP, Fu LL, Jiang CH, Xu YF, Wang X, Yu J: Retina Is Protected by Neuroserpin from Ischemic/Reperfusion-Induced Injury Independent of Tissue-Type Plasminogen Activator. Plos One 2015, 10(7).
18. Li W, Asakawa T, Han S, Xiao B, Namba H, Lu C, Dong Q, Wang L: Neuroprotective effect of neuroserpin in non-tPA-induced intracerebral hemorrhage mouse models. Bmc Neurology 2017, 17.
19. Wu J, Echeverry R, Guzman J, Yepes M: Neuroserpin Protects Neurons from

Ischemia-Induced Plasmin-Mediated Cell Death Independently of Tissue-Type Plasminogen Activator Inhibition. American Journal of Pathology 2010, 177(5):2576-2584.

20. Yepes M, Sandkvist M, Wong MKK, Coleman TA, Smith E, Cohan SL, Lawrence DA: Neuroserpin reduces cerebral infarct volume and protects neurons from ischemia-induced apoptosis. Blood 2000, 96(2):569-576.

21. Armstead WM, Riley J, Cines DB, Higazi AA: PAI-1-derived peptide EEIIMD prevents hypoxia/ischemia-induced aggravation of endothelin- and thromboxane-induced cerebrovasoconstriction. Neurocrit Care 2014, 20(1):111-118.

22. Yang D, Sun YY, Nemkul N, Baumann JM, Shereen A, Dunn RS, Wills-Karp M, Lawrence DA, Lindquist DM, Kuan CY: Plasminogen activator inhibitor-1 mitigates brain injury in a rat model of infection-sensitized neonatal hypoxia-ischemia. Cereb Cortex 2013, 23(5):1218-1229.

23. Yang D, Sun YY, Lin X, Baumann JM, Warnock M, Lawrence DA, Kuan CY: Taming neonatal hypoxic-ischemic brain injury by intranasal delivery of plasminogen activator inhibitor-1. Stroke 2013, 44(9):2623-2627.

24. Yang D, Nemkul N, Shereen A, Jone A, Dunn RS, Lawrence DA, Lindquist D, Kuan C-Y: Therapeutic Administration of Plasminogen Activator Inhibitor-1 Prevents Hypoxic-Ischemic Brain Injury in Newborns. Journal of Neuroscience 2009, 29(27):8669-8674.

25. Nagai N, Suzuki Y, Van Hoef B, Lijnen HR, Collen D: Effects of plasminogen activator inhibitor-1 on ischemic brain injury in permanent and thrombotic middle cerebral artery occlusion models in mice. J Thromb Haemost 2005, 3(7):1379-1384.

26. Krakovsky M, Polianski V, Nimrod A, Higazi A, Leker RR, Lamensdorf I: THR-18, a 18-mer peptide derived from PAI-1, is neuroprotective and improves thrombolysis by tPA in rat stroke models. Neurol Res 2011, 33(9):983-990.

27. Huang X, Ding J, Li Y, Liu W, Ji J, Wang H, Wang X: Exosomes derived from PEDF modified adipose-derived mesenchymal stem cells ameliorate cerebral ischemia-reperfusion injury by regulation of autophagy and apoptosis. Experimental Cell Research 2018, 371(1):269-277.

28. Michalski D, Pitsch R, Pillai DR, Mages B, Aleithe S, Grosche J, Martens H, Schlachetzki F, Haertig W: Delayed histochemical alterations within the neurovascular unit due to transient focal cerebral ischemia and experimental treatment with neurotrophic factors. Plos One 2017, 12(4).
29. Riabinska A, Zille M, Terzi MY, Cordell R, Nieminen-Kelhae M, Klohs J, Pina AL: Pigment Epithelium-Derived Factor Improves Paracellular Blood-Brain Barrier Integrity in the Normal and Ischemic Mouse Brain. Cellular and Molecular Neurobiology 2020, 40(5):751-764.
30. Zille M, Riabinska A, Terzi MY, Balkaya M, Prinz V, Schmerl B, Nieminen-Kelhae M, Endres M, Vajkoczy P, Pina AL: Influence of Pigment Epithelium-Derived Factor on Outcome after Striatal Cerebral Ischemia in the Mouse. Plos One 2014, 9(12).
31. Pillai DR, Shanbhag NC, Dittmar MS, Bogdahn U, Schlachetzki F: Neurovascular protection by targeting early blood-brain barrier disruption with neurotrophic factors after ischemia-reperfusion in rats. Journal of Cerebral Blood Flow and Metabolism 2013, 33(4):557-566.
32. Kwon SK, Ahn M, Song H-J, Kang SK, Jung S-B, Harsha N, Jee S, Moon JY, Suh K-S, Lee SD et al: Nafamostat mesilate attenuates transient focal ischemia/reperfusion-induced brain injury via the inhibition of endoplasmic reticulum stress. Brain Research 2015, 1627:12-20.
33. Wang J, Li C, Chen T, Fang Y, Shi X, Pang T, Zhang L, Liao H: Nafamostat mesilate protects against acute cerebral ischemia via blood-brain barrier protection. Neuropharmacology 2016, 105:398-410.
34. Chen T, Wang J, Li C, Zhang W, Zhang L, An L, Pang T, Shi X, Liao H: Nafamostat mesilate attenuates neuronal damage in a rat model of transient focal cerebral ischemia through thrombin inhibition. Scientific Reports 2014, 4.
35. Li C, Wang J, Fang Y, Liu Y, Chen T, Sun H, Zhou X-F, Liao H: Nafamostat mesilate improves function recovery after stroke by inhibiting neuroinflammation in rats. Brain Behavior and Immunity 2016, 56:230-245.
36. Liu Y, Li C, Wang J, Fang Y, Sun H, Tao X, Zhou X-F, Liao H: Nafamostat Mesilate Improves Neurological Outcome and Axonal Regeneration after Stroke in Rats.

Molecular Neurobiology 2017, 54(6):4217-4231.
37. Yanamoto H, Kikuchi H, Okamoto S, Nozaki K: Cerebral vasospasm caused by cisternal injection of polystyrene latex beads in rabbits is inhibited by a serine protease inhibitor. Surg Neurol 1994, 42(5):374-381.
38. Yanamoto H, Kikuchi H, Okamoto S, Nozaki K: Preventive effect of synthetic serine protease inhibitor, FUT-175, on cerebral vasospasm in rabbits. Neurosurgery 1992, 30(3):351-356; discussion 356-357.
39. Eser C, Kalkan E, Cosar M, Buyukbas S, Avunduk MC, Aslan A, Kocabas V: The effect of aprotinin on brain ischemic-reperfusion injury after hemorrhagic shock in rats: An experimental study. Journal of Trauma-Injury Infection and Critical Care 2007, 63(2):373-378.
40. Homi HM, Sheng H, Arepally GM, Mackensen GB, Grocott HP: Aprotinin Improves Functional Outcome but Not Cerebral Infarct Size in an Experimental Model of Stroke During Cardiopulmonary Bypass. Anesthesia and Analgesia 2010, 111(1):38-45.
41. Sung JH, Zhao H, Roy M, Sapolsky RM, Steinberg GK: Viral caspase inhibitor p35, but not crmA, is neuroprotective in the ischemic penumbra following experimental stroke. Neuroscience 2007, 149(4):804-812.

Supplementary 7. Quality of preclinical studies

Risk of bias assessment

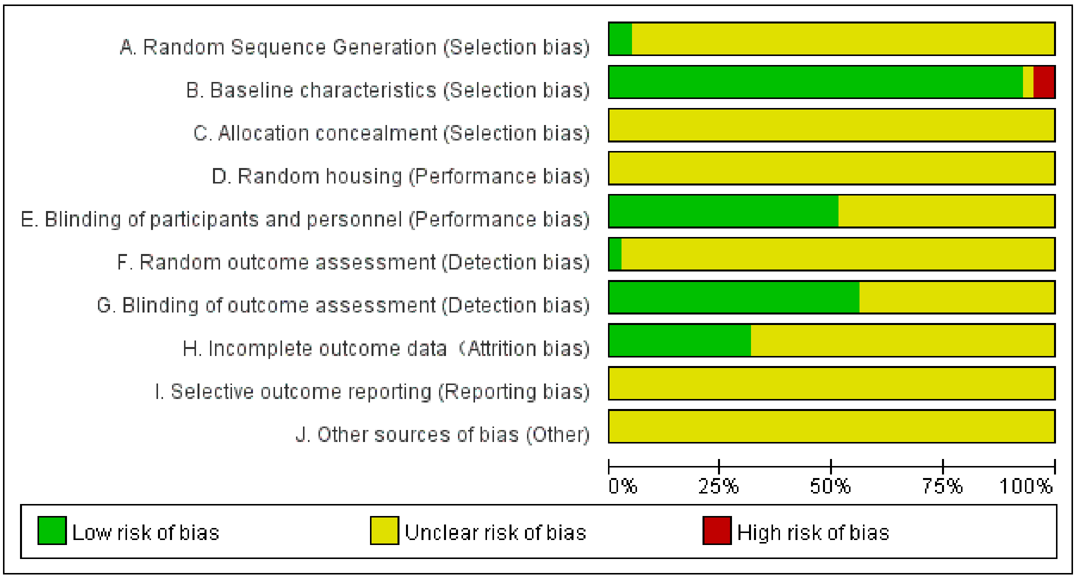

Risk of bias scores of 41 animal studies included

	A. Random Sequence Generation (Selection bias)	B. Baseline characteristics (Selection bias)	C. Allocation concealment (Selection bias)	D. Random housing (Performance bias)	E. Blinding of participants and personnel (Performance bias)	F. Random outcome assessment (Detection bias)	G. Blinding of outcome assessment (Detection bias)	H. Incomplete outcome data （Attrition bias)	I. Selective outcome reporting (Reporting bias)	J. Other sources of bias (Other)
Akita (2003)	?	+	?	?	?	?	?	?	?	?
Armstead (2014)	?	+	?	?	?	?	?	?	?	?
Buchthal (2018)	?	+	?	?	?	?	?	?	?	?
Chen (2015)	?	+	?	?	?	?	?	?	?	?
Chen (2018)	+	+	?	?	+	?	+	+	?	?
Cuomo (2007)	?	+	?	?	+	?	+	?	?	?
De Simoni (2003)	?	+	?	?	+	?	+	+	?	?
De Simoni (2004)	?	+	?	?	+	?	+	?	?	?
Eser (2007)	?	+	?	?	?	?	+	?	?	?
Gesuete (2009)	?	+	?	?	+	?	+	?	?	?
Gu (2015)	?	+	?	?	?	?	?	?	?	?

Heimann (1999)	?	+	?	?	?	?	?	?	?	?
Heydenreich (2012)	?	-	?	?	+	?	+	?	?	?
Homi (2010)	?	+	?	?	+	?	+	?	?	?
Huang (2018)	?	+	?	?	+	?	+	?	?	?
Krakovsky (2011)	?	+	?	?	+	?	+	+	?	?
Kwon (2015)	?	+	?	?	?	?	?	+	?	?
Li (2016)	?	+	?	?	?	?	?	+	?	?
Li (2017)	?	+	?	?	+	?	+	?	?	?
Liu (2017)	?	+	?	?	+	+	+	+	?	?
Mercurio (2021)	?	+	?	?	+	?	+	+	?	?
Michalski (2017)	?	+	?	?	+	?	+	+	?	?
Moldthan (2014)	?	+	?	?	?	?	+	?	?	?
Nagai (2005)	?	+	?	?	?	?	?	?	?	?
Pillai (2013)	?	+	?	?	+	?	+	+	?	?
Riabinska (2020)	?	+	?	?	+	?	+	+	?	?
Storini (2004)	?	+	?	?	+	?	+	?	?	?
Sung (2007)	?	+	?	?	?	?	?	?	?	?
Tomasi (2011)	+	+	?	?	+	?	+	+	?	?
Vollmer (1989)	?	+	?	?	?	?	?	?	?	?
Wang (2016)	?	+	?	?	?	?	?	?	?	?
Weaver (2002)	?	+	?	?	?	?	?	?	?	?
Wu (2010)	?	+	?	?	?	?	?	+	?	?
Yanamoto (1992)	?	+	?	?	+	?	+	?	?	?
Yanamoto (1994)	?	+	?	?	+	?	+	?	?	?
Yang (2009)	?	+	?	?	?	?	?	?	?	?
Yang (2013a)	?	?	?	?	?	?	?	?	?	?
Yang (2013b)	?	-	?	?	?	?	?	?	?	?
Yepes (2000)	?	+	?	?	?	?	?	?	?	?
Zhang 2021	?	+	?	?	+	?	+	?	?	?
Zille (2014)	?	+	?	?	+	?	+	+	?	?

Supplementary 8. Publication bias of preclinical studies

As shown by the asymmetry of funnel plots, there was publication bias in the studies of C1-INH in MCAO animals while no publication bias was revealed in the studies of FUT175.

Each blue dot indicated an experiment. Red line indicated the overall effect size. Grey line showed 95%CI. The asymmetry of funnel plot suggested publication bias.

A. Studies of C1-INH in MCAO models

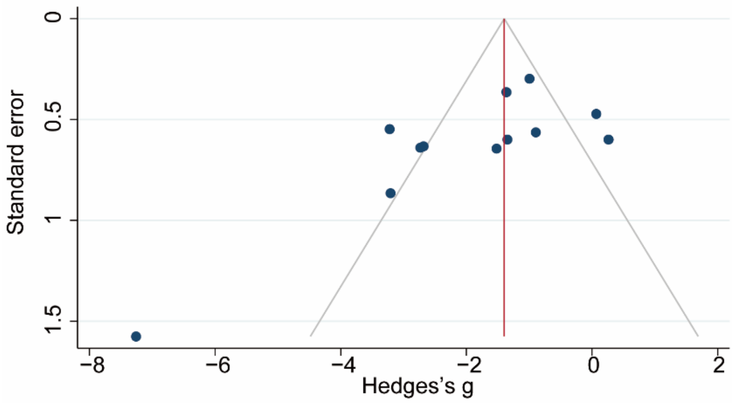

B. Studies of FUT175 in MCAO models

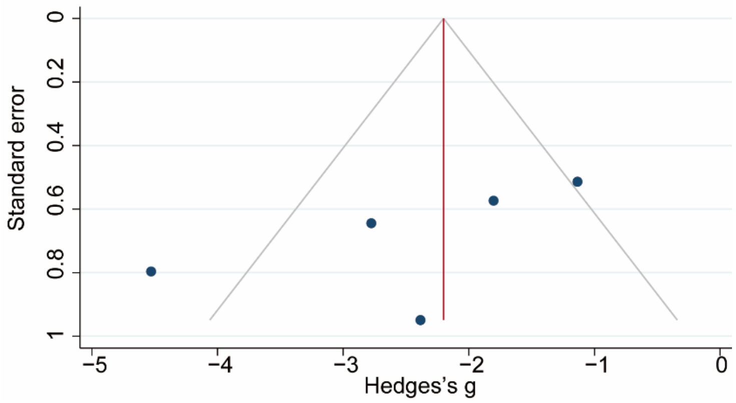

Supplementary 9. Sensitivity analysis of preclinical studies

Leave-one-out method was used to assess the influence of a single study on the overall effect and the robustness of meta-analysis. One study was omitted and meta-analysis was performed on the remaining studies. The Hedges's g of leave-one-out analysis fell into the 95% CI of the orginal effect size.

Study ID indicated the removed study. Red lines indicated the overall effect of all studies included and the 95%CI.

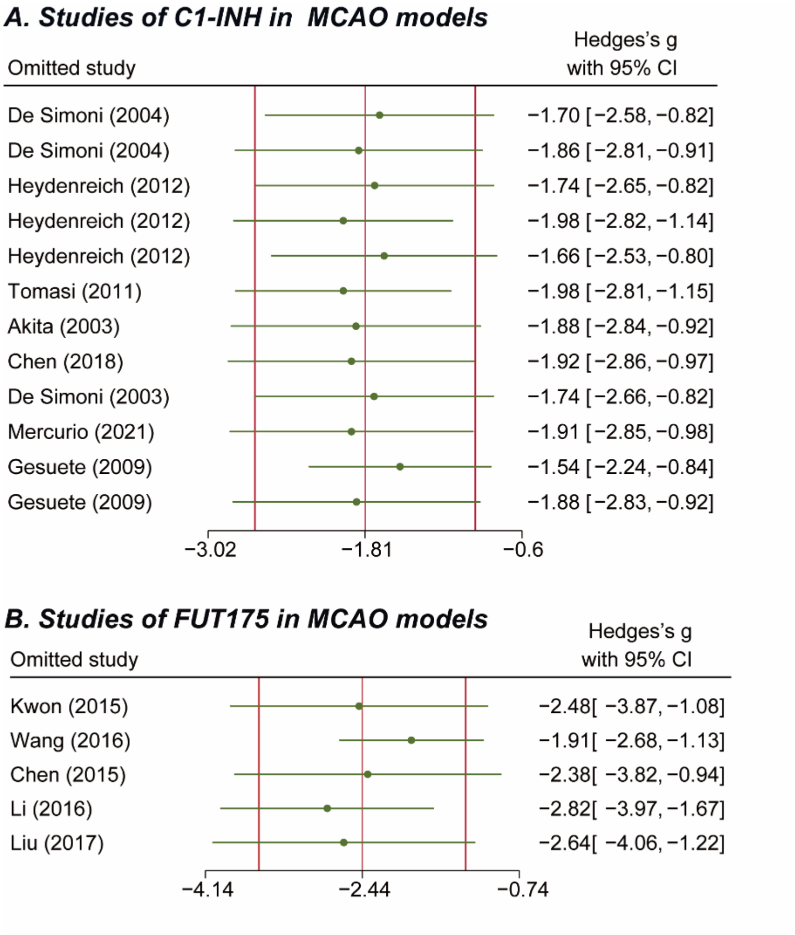

Supplement: Supplementary file 1 — Appendix S1 [file CNS-29-1738-s002.pdf]
